# Plasma Electrolytic Modification of Zirconium and Its Alloys: Brief Review

**DOI:** 10.3390/ma16165543

**Published:** 2023-08-09

**Authors:** Boris L. Krit, Andrey V. Apelfeld, Anatoly M. Borisov, Natalia V. Morozova, Alexander G. Rakoch, Igor V. Suminov, Sergey N. Grigoriev

**Affiliations:** 1 Department of Devices and Information Systems Production for Aircraft Control, Moscow Aviation Institute (National Research University), Moscow 109383, Russia; anatoly_borisov@mail.ru; 2 Department of High-Efficiency Processing Technologies, Moscow State University of Technology “STANKIN”, Moscow 127994, Russia; apelfeld@yandex.ru (A.V.A.); innat.m@mail.ru (N.V.M.); ist3@mail.ru (I.V.S.); s.grigoriev@stankin.ru (S.N.G.); 3 Department of Medical Technique, Russian Medical Continuous Professional Education Academy, Moscow 123995, Russia; 4 Department of Corrosion, National University of Science and Technology (NUST) Moscow Steel and Aloys Institute (MISIS), Moscow 119049, Russia; rakoch@mail.ru

**Keywords:** zirconium, zirconia, plasma electrolytic modification, properties, coating

## Abstract

The review focuses on the surface modification of Zr and its alloys, which is necessary to expand the applications of these kinds of materials. Data on the properties of pure zirconium and its alloys are presented. Since surface engineering and the operation of the above materials are in most cases associated with the formation of oxide coatings, information on the characteristics of ZrO_2_ is given. In addition, attention is paid to phasing in the zirconium–oxygen system. It is noted that the most effective method of surface engineering of Zr and its alloys is plasma electrolytic modification (PEM) technology. Specific examples and modes of modification are described, and the reached results are analyzed. The relevance, novelty and originality of the review are determined by the insufficient knowledge about a number of practical features concerning the formation of functional oxide coatings on Zr and some of its alloys by the technology of PEM. In particular, the information on the phase composition and possibilities of stabilization of the tetragonal and cubic modifications of ZrO_2_, the effects of the component composition of electrolyte solutions and electrolyte suspensions, and the specifics of the treatment of additive shaping and deformed materials are rather contradictory. This review aims to collect recent advances and provide insights into the trends in the modification of Zr and its alloys, promote the formulation of practical recommendations and assess the development prospects.

## 1. Introduction

Zirconium and its alloys have high physical–mechanical and physical–chemical properties compared with other structural materials. For this reason, they are widely used in many industrial fields from airspace to medicine, and especially in the nuclear industry. However, such characteristics as high electrical conductivity, relatively low hardness, insufficient tribomechanical characteristics and corrosion resistance in many operating environments significantly limit the area of practical implementation of zirconium materials. Nevertheless, it should be noted that there is a tendency for more and more active consumption of zirconium alloys. Due to their low thermal conductivity, heat resistance and absence of cold brittleness, they are widely demanded for products of aviation and rocket and space technology [1,2]. Zirconium, in particular, alloyed with Nb, Ti, Ta, Hf, etc., is used in medicine due to its high mechanical properties due to solid solution hardening. These alloys have a complex of properties relevant for such applications (bi inertness and biocompatibility, low thermal conductivity, high fatigue strength and cyclic durability) [3,4,5,6], which makes them very attractive, for example, when used as dental implants. However, in this case, one of the most important problems is corrosion processes since the body’s environment is highly aggressive. The active metabolism of bacteria in the oral cavity leads to a local decrease in the pH value up to an acidic environment, and the use of toothpastes and rinses containing fluorides leads to their increased concentration. The presence of hydrofluoric acid can destroy the natural protective oxide layer on the zirconium surface. To prevent such undesirable actions on the surface of implants, it is advisable to apply coatings that have anticorrosive protective properties, on the one hand, and biologically active and biocompatible properties, on the other [7].

Zirconium-based compositions have an important role in nuclear power engineering since they have a low thermal neutron and electron capture cross-section in combination with high ductility. Despite the significant drawback of the interaction with water vapor and the formation of hydrides diffusing into the alloy, zirconium alloys are practically indispensable for fuel rods, core-clad and guide tubes, grids, pressure pipes and exhaust vessels of nuclear reactors [8,9]. At the same time, the vast majority of information sources are unanimous in assessing the corrosion resistance of Zr as insufficient and contain imperative recommendations for finding effective ways to protect against the negative impact of the environmental media.

Recently, extensive ways of development for applied materials science have practically completely exhausted themselves, giving way to the search for methods of modification (i.e., changes towards improvement) of the properties of materials and products. Preference is given to surface treatment because in most cases, the exact characteristics of the surface determine the level of properties of the product as a whole. Surface engineering is a relatively new direction in science and technology, including both traditional and innovative methods of modification.

It covers many areas of modern materials science and is based on the processes of formation of surface layers with the required properties directly on the base material. Often the outer layers of other, usually composite, materials with properties different from those of the substrate are formed by various methods. In addition, surface modification seems to be more attractive in comparison with methods of changing the bulk characteristics of materials and products from an economic standpoint [10,11].

All of the above has led to the development of scientific research and technological developments aimed at improving the traditional methods and creating new methods of influencing the surface in order to give it the parameters set by the operating conditions. This approach allows the most effective use of the combination of properties of the base material and the modified layer; in addition, surface treatment operations can be easily integrated into the technological process of manufacturing or repairing products. The creation and implementation of new environmentally friendly technologies for modifying surfaces are currently one of the key areas of contemporary science and technology.

For the formation of products from various materials, additive technologies have been actively introduced in recent years. Their main advantages are a significant reduction in both the consumption of materials and production waste, which entails significant cost savings. Furthermore, there is a possibility of product variability and individualization, as well as the manufacturing of parts of high complexity with improved characteristics. Additive methods of shaping also could be applicable to zirconium alloys and compounds [12].

Like other materials, Zr and its alloys are of interest as an object for surface engineering technologies. A large number of studies related to this scientific direction are known. An analysis of publications shows that the main way should be considered the formation of surface multifunctional ceramic-like (most often, oxide) coatings [1,13,14,15,16,17,18,19,20,21,22,23,24,25,26]. At the same time, most authors point out that plasma treatment in electrolytes is preferable for obtaining oxide-ceramic surface layers.

It is known that plasma electrolytic modification (PEM) entails a substantial increase in widely demanded materials performance indicators such as mechanical properties, corrosion resistance, tribological properties and thermophysical properties [11,27,28,29,30]. This certainly is also applicable to zirconium materials. It follows from the works published recently that besides improvement of the above-mentioned technical properties, PEM is capable of giving Zr alloys unique specific abilities such as superhydrophobicity [31], biomedical abilities [21,32], optical (luminescent and photocatalytic) abilities [22,33,34,35,36,37,38], crack resistance [39,40,41] and resistance to ionizing radiation [42].

The relevance, novelty and originality of the review are determined by the insufficient knowledge about a number of practical features concerning the formation of functional oxide coatings on Zr and some of its alloys by the technology of PEM. In particular, the information on the phase composition and possibilities of stabilization of the tetragonal and cubic modifications of ZrO_2_, the effects of the component composition of electrolyte solutions and electrolyte suspensions, and the specifics of the treatment of additive shaping and deformed materials are rather contradictory. The purpose of this review is to make a feasible contribution to the systematization and generalization of the available theoretical and applied information, to analyze the information about the modifying of Zr and its alloys, to formulate practical recommendations and to assess the development prospects. To this end, the review also includes data on the properties of Zr and some of its alloys, as well as useful information on the properties of ZrO_2_. In addition, methods for the surface engineering of zirconium alloys by applying zirconium oxide coatings are described, with emphasis on PEM technologies.

## 2. Basic Properties of Zr

Zirconium ranks 21st in terms of the abundance of elements in the Earth’s crust; its crustal content (0.025%) exceeds the content of such metals widely used in engineering as nickel, zinc, copper, tin, lead and cobalt [43].

Zirconium is a shiny silver-gray metal existing in three crystalline modifications: α, β and ω:

α-Zr—hexagonal system, space group P6_3_/mmc, cell parameters a = 0.3231 nm, c = 0.5146 nm, Z = 2, ρ = 6.5107 g/cm^3^ with a close-packed lattice of the magnesium type.

β-Zr—cubic system (body-centered lattice), space group Im_3_m, cell parameters a = 0.361 nm, Z = 2 with a lattice of the α-Fe type. The α ↔ β transition occurs at 863 °C, and the ΔH of the transition is 3.89 kJ/mol. The additions of Al, Sn, Pb and Cd increase the transition temperature, and Fe, Cr, Ni, Mo, Cu, Ti, Mn, Co, V and Nb decrease it.

ω-Zr is a metastable hexagonal phase arising at high pressures; it is not close-packed and has three atoms per unit cell [44,45].

Pure zirconium is plastic, easily amenable to cold and hot processing (rolling, forging, stamping). The presence of even small amounts of oxygen, nitrogen, hydrogen and carbon (or compounds of these elements with zirconium) dissolved in the metal causes the fragility of zirconium. It has the following properties: elastic modulus (20 °C), 97 GN/m^2^; tensile strength, 253 MN/m^2^; Brinell hardness, 640–670 MN/m^2^. The oxygen content has a very strong effect on hardness: at a concentration of more than 0.2%, zirconium does not lend itself to cold working. The strength characteristics of Zr and its alloys strongly depend on the methods of preparation and composition. As follows from the data in Table 1 [43], the differences can be more than 2-fold.

In [46], a number of physical and mechanical characteristics of zirconium alloy grade 702 (Zr-4.5 Hf) are given. Its strength (σ_B_ = 380 MPa; σ_0.2_ = 207 MPa) and plasticity (δ = 16%) are inferior to those of alloys of the Zr-Nb system. The thermal conductivity of pure Zr and most of its industrially significant alloys under standard conditions is 22.6 W/(m·K) [46,47].

Additional types of treatment can significantly improve the mechanical characteristics of zirconium alloys [9,48,49,50]. Improvement of properties, as a rule, is associated with the refinement of the structure down to the nanoscale state.

As already noted, zirconium alloys have a relatively small thermal neutron capture cross-section (0.185 b) [5,6], greater than that of beryllium (0.09 b) and magnesium (0.059 b) but lower than that of aluminum (0.2158 b).

Chemically, zirconium is very active; its ionization potential is 6.84 eV, and its electronegativity is 1.1 [10]. Zr is characterized by an oxidation state of +4; lower oxidation states of +2 and +3 are known only for zirconium compounds with chlorine, bromine and iodine.

Bulk compact zirconium begins to oxidize slowly in the range 200–400 °C, becoming covered with a film of zirconium dioxide ZrO_2_; above 800 °C, it vigorously interacts with atmospheric oxygen. Powdered metal is pyrophoric—it can ignite in air at normal temperatures. Zirconium actively absorbs hydrogen already at 300 °C, forming a solid solution and hydrides ZrH and ZrH_2_. However, at 1200–1300 °C in a vacuum, hydrides dissociate, and all hydrogen can be removed from the metal. With nitrogen, zirconium forms ZrN nitride at 700–800 °C. Zirconium reacts with carbon at temperatures above 900 °C to form ZrC carbide. Zirconium carbide and zirconium nitride are hard refractory compounds. Zirconium reacts with fluorine at ordinary temperatures, and with chlorine, bromine and iodine at temperatures above 200 °C, forming the higher halides ZrHal_4_ (where Hal is halogen).

Zirconium is stable in water and water vapor up to 300 °C; at higher temperatures, (starting from about 700 °C) an exothermic vapor–zirconium reaction begins [51]:

Zr + 2H_2_O → ZrO_2_ + 2H_2_ ↑

Zirconium and its alloys, as a rule, do not react with hydrochloric and sulfuric (up to 50%) acids or with alkali solutions, although they dissolve in hydrofluoric and hot concentrated (above 50%) sulfuric acids. They interact with nitric acid and “aqua regia” (mixture of nitric acid and hydrochloric acid in an optimal molar ratio of 1:3) at temperatures above 100 °C.

Impurities and alloying elements have the following effect on the corrosion resistance of zirconium [43,52]:

(1) H, N, C, O, Ti, Al Ca, Mg, Cl, Si Pb, Mo, Zn, La, Ce, Ga, V, Be and Ta accelerate corrosion;
(2) Sn, Sb, Fe, Cr and Ni weaken the harmful effect of the elements of the first group;
(3) Hf, Y, Cu and W are neutral.

The balance of properties determines the areas of usage of zirconium materials. Metallic zirconium is applied mainly in the nuclear power industry and in the production of equipment for the chemical industry. Since metallic zirconium and its insoluble compounds (dioxide, silicate) have a sufficiently high biological inertness with respect to tissues and body fluids, an increase in the use of these materials in biomedicine has been noticeable in recent years. Zirconium does not induce any noticeable reaction in muscle tissue, bone or brain; therefore, it can be used for the manufacture of medical invasive devices [43]. The combination of passivation, corrosion resistance and satisfactory biotolerance to bone progenitors makes the Zr-based alloys promising implant materials [53,54].

At the same time, the disadvantages of zirconium and zirconium alloys as structural materials include the following [43]:

1. Relatively low mechanical properties, especially at temperatures above 300 °C.
2. Insufficient corrosion resistance:
  - in water and steam at 300 °C or more;
  - in dry and especially humid air and carbon dioxide from 500 to 550 °C.
3. Embrittlement when saturated with atmospheric gases (nitrogen and oxygen).
4. Presence of allotropic transformation at 863 °C.
5. Low temperature of the beginning of recrystallization (from 500 °C), leading to softening.

The usage of zirconium and its alloys in aggressive environments results in shorting of service life [54]. This fact is very relevant for nuclear materials and other industries. To improve the performance properties, as well as to prevent undesirable effects, it is advisable to apply coatings on the surface of materials that have a modifying and protective effect. Many researchers agree that the formation of Zr-oxide base coatings is practically the best option for raising the complex of properties of products from zirconium and its alloys to a higher level [1,9,11,13]. In oxygen-containing media, an oxide chemisorption film several atomic layers thick is formed on the surface of zirconium which nevertheless has a high passivating ability [55]. This logically and naturally follows from the characteristics of the oxide itself and some of its isomorphism to the metal. As a result, natural surface oxide phases, which are an integral part of real objects, have a tangible effect on the integral properties. This applies even more for specially created oxide-ceramic surface layers and coatings. Therefore, it seems appropriate to analyze the characteristics of the most likely and thermodynamically stable compound in the zirconium–oxygen system.

## 3. Basic Properties of ZrO_2_

Zirconium oxide or zircon (chemically dioxide ZrO_2_) is a colorless crystalline substance, one of the most refractory metal oxides (T_m_ = 2715 °C). It exhibits amphoteric properties; it is insoluble in water and aqueous solutions of most acids and alkalis, but it dissolves in hydrofluoric acid and concentrated sulfuric acid with the addition of ammonium sulfate upon prolonged heating. It interacts with alkali melts [56].

The physical and mechanical properties of ZrO_2_ (sintered ceramics, residual porosity <0.1%) are presented in Table 2 [57,58,59,60].

Compared with alumina ceramics (α-Al_2_O_3_), zirconia is 1.5 times heavier and has almost half the hardness. Moreover, its strength and fracture toughness are several times higher, and the thermal conductivity is an order of magnitude lower [58].

For a long time, it was believed that zirconium dioxide exists in three crystalline forms [56,61,62,63]. Under normal conditions, zirconium dioxide crystallizes at 390 °C in a stable monoclinic form (*m*-ZrO_2_, density 5.56 g/cm^3^), which occurs naturally in the form of the mineral baddeleyite. In 1929, C. Ruff and F. Ebert established that at a temperature of about 1100 °C, the formation of a metastable medium-temperature tetragonal phase (*t*-ZrO_2_, density 6.1 g/cm^3^) occurs, which is present in many zirconium ceramics. Above 2300 °C, up to the melting point (2715 °C), undoped ZrO_2_ is characterized by an unstable high-temperature cubic modification (*c*-ZrO_2_, density 6.27 g/cm^3^). In recent years, a number of studies have provided evidence of the presence in the temperature range of 2300–2500 °C of the second tetragonal allotropic form (*t*′-ZrO_2_) with a low degree of tetragonality, the existence of which was first reported by T. Noguchi and H.G. Scott [64,65,66]. The intermediate *t*′-phase is structurally identical to *t*-ZrO_2_; however, a change in the volume of the tetragonal cell with increasing temperature entails a decrease in the degree of tetragonality down to unity, i.e., transformation into a cubic modification structure.

**Figure 1:**
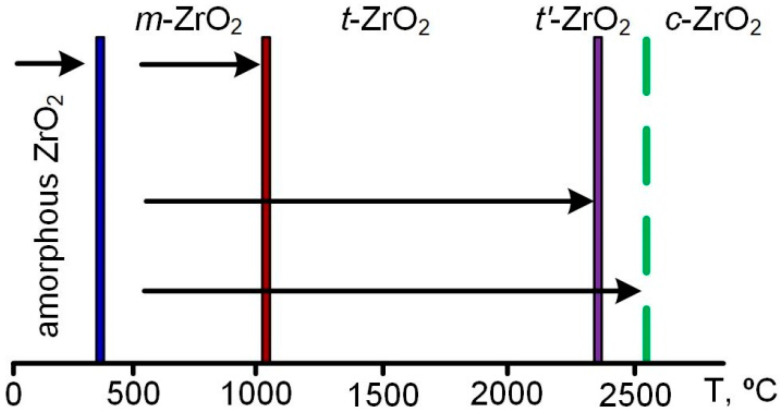
Temperature ranges of the existence of ZrO_2_ phases.

All phase transitions (Figure 1) are diffusion-less martensitic transformations, which can be represented by the scheme (*m* ↔ *t* ↔ *c*) or (*m* ↔ *t* ↔ *t*′↔ *c*). In this case, the tetragonal phase is rather difficult, and the cubic shape is practically impossible, to maintain at room temperature by quenching. Cubic ZrO_2_ is the highest-temperature phase and has the highest density, but it is also the most unstable because of the strong distortion (curvature) of the oxygen sublattice [63]. High-temperature phases are usually stabilized by ultrafast heat treatment or the introduction of additives such as MgO, CaO and Y_2_O_3_.

On the other hand, the transition of the cubic and tetragonal phases of zirconium dioxide to the monoclinic one is accompanied by an increase in volume, which improves the strength of the ceramic. A local increase in volume (and the corresponding pressure) leads to the appearance of mechanical stresses at the tops of growing microcracks and, as a consequence, stabilizes them and slows down further growth [56]. The authors of [14] believe that the optimal phase composition for zircon ceramics is the maximum content of the tetragonal *t*′-phase in the presence of an insignificant (4–5 wt.%) fraction of the monoclinic phase. The tetragonal phase provides a high degree of thermal stability, and due to the martensitic transformation of the monoclinic phase, a network of small cracks is formed that prevents fracture.

## 4. Formation Oxide-Ceramic Surface Layers on Zr

Various modification methods are applied to improve the surface properties with coat formation. For zirconium materials, the most commonly applicable are PVD and/or CVD deposition methods (including gas plasma spraying of coatings; electron-beam sputtering of targets; ion-plasma, magnetron and electric arc spraying; electrospark alloying), as well as thermal oxidation methods [1,14,15,16,17,18,20].

### 4.1. Vacuum-Deposited Coatings

The work [14] investigated multilayer heat-shielding plasma coatings (HPCs) designed to protect parts of internal combustion engines. ZrO_2_ powder stabilized with 6.2 wt.% Y_2_O_3_ was used for spraying; plasma coatings were applied using a UPU-8M setup. Steel samples were used as a basis for the coating, and in the study of heat-shielding properties, samples of aluminum alloy were used; NiCrAlY alloy served as a sublayer. Spraying mode was U = 40–60 V, I = 500 A; plasma-forming gas consumption (N_2_ or N_2_ + Ar mixture) was 24–31 L/min, and spraying distance was 100–130 mm.

The thickness of the ceramic coating for all samples was approximately the same and in the amount of about 200 μm, and the thickness of the NiCrAlY sublayer was from 50 to 150 μm. There was no lamellarity in the formed layer structure, and its average microhardness was at a level of about HV11000 MPa. The authors note that the thickness of the metal sublayer has practically no effect on the structure and microhardness of the ZrO_2_ surface layer; however, it had an influence on the phase composition. When the metal sublayer had a thickness of 50 and 100 μm, the main phase of the ceramic layer was *t*-ZrO_2_, and the mass share of the monoclinic phase decreased to 2% (in the initial powder used for deposition, the content was 10.2 wt.%). At a sublayer thickness of 150 μm, in addition to the listed phases, about 12 wt.% was the cubic modification *c*-ZrO_2_.

The phase composition of the ceramic coating layer at a depth of 100 μm from the surface is almost the same: tetragonal with 4 wt.% monoclinic.

Assessment of the tetragonality degree and volume of the unit cell of the tetragonal phase ZrO_2_ showed that with a sublayer thickness of 150 μm, the degree of tetragonality of the ZrO_2_ (the ratio of the lattice parameters c/a) is 1.0113, and the volume of the unit cell is 134.5793·10^−3^ nm^3^. With a sublayer thickness of 50 and 100 μm, a phase is formed with a lower degree of tetragonality (c/a = 1.0107 and 1.0108) and an increased volume of the unit cell (134.6823·10^−3^ and 134.7030·10^−3^ nm^3^, respectively). In structure, these phases approach the quenched phase of *t*′-ZrO_2_, which was stable in a wide temperature range and at a cyclic change in temperature.

Direct flame heating with a gas burner was used to study the heat-shielding properties of the coatings. The burner was placed at a distance of 35–40 mm from the surface of the coated sample. The samples were heated for 3 s to the temperature of 400 °C and then cooled to a temperature of 70–80 °C for about 6 s. The thermal cycling was carried out until the coating was broken or peeled off from the base by no more than 15%.

During the heat resistance test, samples with two-layer coatings survived 1500 thermal cycles, while no external changes were detected and their microhardness practically did not change compared with the initial state. Only after 2000 heat cycles of tests did a longitudinal crack appear in the zone of connection with the sublayer, which spread into the ZrO_2_ layer and caused its minor destruction.

To reveal the dynamics of heating, from the opposite side of the sample, a thermocouple was fixed at a depth of 2 mm. The temperature rises of the substrate without coating as well as with two-layer coating were recorded. Up to 10 heating and cooling cycles were carried out. By analyzing the cycloramas, it was found that the maximum heating temperature of the samples with HPC decreases from 415 (uncoated) to 365 °C; i.e., the coating reduces the temperature of the aluminum base by 50 °C.

The authors of [15] presented data on the optimization of the processes for obtaining the maximum content of the tetragonal phase in the initial material and in heat-shielding coatings based on zirconium dioxide, obtained using the APS method (atmospheric plasma spraying or air plasma spraying). The formation of the HfO_2_–ZrO_2_–Y_2_O_3_ oxide system, which is a microstructure similar to zirconium dioxide, transformed for use at temperatures of 1300 °C, is described. The mechanism of the influence of hafnium oxide on the microstructure formation is explained. Plasma spraying (especially APS) has a number of advantages over other coating methods, but it should be noted that it tends to form porous coatings. Therefore, these technologies are suitable for surface modification of materials exposed to short-term exposure to low-corrosive environments.

Plasma coatings containing ZrO_2_, more than other oxide systems, contribute to the complex ennoblement of mechanical properties. For example, in [67], the results of impact loading tests using the Charpy method on samples of 2024 aluminum alloy are described. Two types of coatings were deposited by APS: ZrO_2_/20%Y_2_O_3_ and Al_2_O_3_. To achieve a good adherence between the coatings and substrate, a bond coat of NiCr was also deposited by APS. The fracture energy of the Al_2_O_3_-coated samples was found to be 3.43% higher compared to the uncoated ones, whereas the same parameter for the ZrO_2_/20%Y_2_O_3_-coated samples was 18.28% higher. At the same time, during the impact tests, uncoated and ZrO_2_/20%Y_2_O_3_-coated samples showed a plastic failure behavior in contrast to Al_2_O_3_-coated samples, which showed a brittle failure behavior.

In [17], the preparation by magnetron RF-sputtering of a ceramic target for nanostructured coatings of stabilized zirconium dioxide Zr(Y,Hf)O_2_ with a two-phase structure consisting of monoclinic and tetragonal phases is presented. A feature of the method is, firstly, the possibility of forming nanostructured layers, which should lead to an improvement in mechanical properties (in particular, hardness and strength) in accordance with the Hall–Petch empirical law. Second, the presence of a large volume fraction of grain boundaries should lead to a decrease in the phonon thermal conductivity, which is a significant positive factor. It was found that the partial pressure of oxygen O_2_ in the working chamber affects the phase ratio; in particular, an increase in the O_2_ pressure (from 0.23 to 0.65 Pa) leads to a significant increase in the volume fraction of the monoclinic phase in the sprayed coating (from 15 to 85%). It is shown that the grain size of the nanostructure being formed is sensitive to the amount of O_2_ only at low partial pressures (less than 0.35 Pa). The formation of a single-phase (tetragonal) structure in the coating occurs after annealing at a temperature of 1100 °C and higher, while the nanostructuring of the material is preserved. The transition to a single-phase structure is accompanied by an increase in the microhardness of the coating.

At the same time, it is known that the methods of ion-plasma deposition of protective coatings do not provide high thermal cyclic resistance and sufficiently good adhesion.

The results presented in [68] can be considered as a contribution to the solution of this problem. The authors investigated the radiofrequency magnetron deposition of yttrium-stabilized zirconium dioxide films (ZrO_2_ + 8% Y_2_O_3_) on stainless steel AISI 316L (SS), widely used for biomedical purposes. Various power densities (50–250 W) and deposition durations (30–120 min) were used to give the coatings the most favorable crystallographic orientation and morphology. As it was found, the coatings have a (111) preferred orientation during crystal growth along the c-axis for a short deposition time (30–60 min), while a polycrystalline structure is formed during deposition from 90 to 120 min. This made it possible to achieve a significant, at least 46 times, increase in adhesion and corrosion resistance. Coatings formed at 200 W for 120 min also showed a higher fracture potential.

However, it should be noted that the method of magnetron high-frequency sputtering is characterized by greater technological complexity and low productivity.

### 4.2. Chemical–Thermal Oxidation

The formation of surface oxides on zirconium and its alloys is also possible chemically.

**Figure 2:**
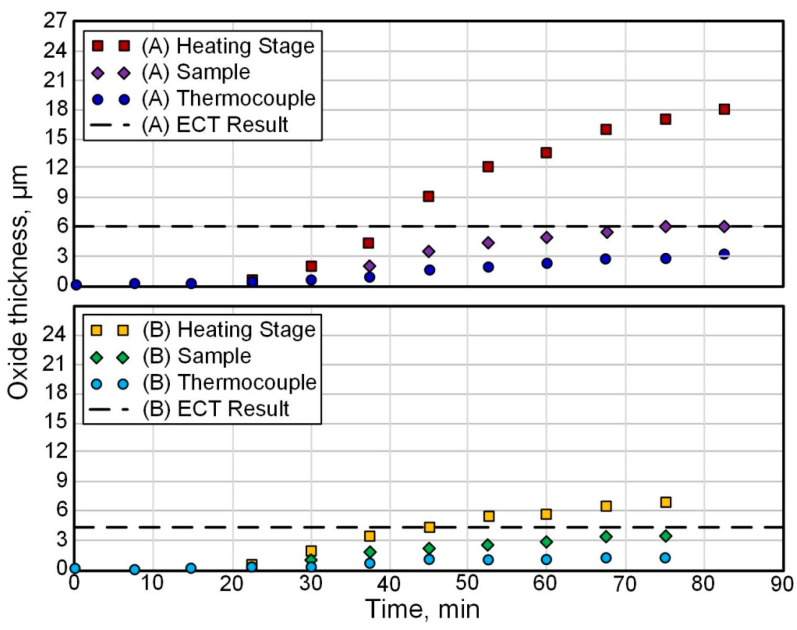
Results of Zircaloy-4 air furnace oxidation [42]. Reprinted with permission from Ref. [42]. 2014, The Authors.

**Figure 3:**
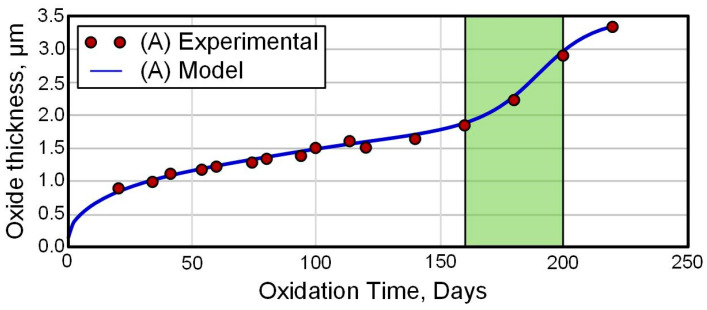
Oxidation kinetics for Zircaloy-4 tested at 360 °C in simulated primary water. The shaded green area identifies transition in the corrosion kinetics [42]. Reprinted with permission from Ref. [42]. 2014, The Authors.

For example, in [42], using the in situ synchrotron X-ray diffraction (S-XRD) technique, an analysis of oxide layers formed on Zircaloy-4 alloys in an air furnace was performed at oxidation temperatures of 650 °C (Test A) and 710 °C (Test B) with a heating rate of ~11.2 °C/min and holding time of ~40 min. The growth kinetics of the oxide layer is shown in Figure 2. The thickness of the oxide was determined based on the measurement of the increase in weight. It can be seen that the increase in the film thickness occurs according to the parabolic law. However, the protective ability of such a coating is a limited resource, as evidenced by ex situ data obtained for a long time in an autoclave at 360 °C (Figure 3).

By S-XRD, it was found that as the oxide thickness increases over time, stress relaxation occurs in both the monoclinic and metastable tetragonal phases. In this case, a decrease in the fraction of the tetragonal phase is noted. It was shown by the finite element method that the effect of creep on stress relaxation in the oxide layer is negligible. This means that relaxation processes are associated with other mechanical degradation mechanisms, such as the development of roughness at the metal–oxide interface or the destruction of the oxide layer itself.

A study was carried out and a comparison was made of the features and kinetics for thermal oxidation of the Zr-2.5Nb alloy obtained by various technological methods (conventional casting, forging, electron-beam melting, hot isostatic pressing) in the temperature range 450–600 °C [24]. It was found that the oxidation kinetics of cast and forged materials obey the parabolic law, while for alloys subjected to hot isostatic pressing and electron-beam melting, parabolic linear dependence is characteristic. The authors attribute the differences to the distinctions in grain size of the structure of the materials, since the large size of the grains increases oxidation. Be that as it may, the oxide layers of all materials mainly consisted of the monoclinic zirconium phase and a minor amount of the tetragonal zirconium phase which transformed from *t*-ZrO_2_ back to *m*-ZrO_2_ with increasing oxidation time. The surface hardness of cast, forged and hot isostatically pressed materials increased from 215, 204 and 188 HV to 902, 1070 and 1137 HV after oxidation, respectively. Analysis of the cross-sections of the materials showed the presence of micropores and microcracks inside oxide layers with a thickness of 4 to 8 μm. The authors conclude that it is possible to obtain a dense layer of black *m*-ZrO_2_ oxide with a smooth surface and a hardness of 902 HV at a temperature of 600 °C and an oxidation time of 3 h.

There are some noteworthy investigations of the impact of chemisorbed fluorine on the surface thermal oxidation of Zr [69,70,71,72,73]. Based on the pseudopotential theory formulated by V. Heine, M. Cohen and D. Weir [74], the influence of impurities according to their period (B, C, N_2_, O_2_, F_2_) and type (B, C, N_2_, O_2_, F_2_) on the scattering of valence conduction-band electrons was studied. According to their model, the increase in a nucleus charge of an impurity results in a rise in the electron scattering at its ionic atomic core. This is equivalent to the increase in the doped ion shielding by conduction-band electrons or to the increase in the charge’s “cap” around the doped ion. The fluorine ion, which has the deepest potential pit, has the highest charged “cap”. Formation of the charged “caps” around an impurity leads to the decrease in electron density in gaps, located between the scattering centers, not as a result of electron number change in the surface metal layer, but as a result of their relocation. The latter circumstance is tantamount to the thermal expansion of the lattice, and in this case the “increase in temperature” in the gaps with smaller electron density. The depth of a potential pit increases with the period and type of the abovementioned impurities. Thus, fluorine gives the deepest potential pit, and the effective scattering of conduction-band electrons increases with the ionic atomic core of the impurity.

A similar mechanism (so-called “screening effect”) leading to the decrease in strength of Me1-Me1, Me1-Me2, etc., atomic bonding at places of a metallic surface, which are free from chemisorbed ions, was proposed by V.A. Tskhai and P.V. Gel’d [75]. The screening effect is based on the system energy gain, which takes place previously and occurs as a result of the electrostatic repulsion between the electrons of the fluorine ion and the electrons providing interatomic bonding in the surface metallic layer. This leads to the breaking of this bonding. Therefore, the system energy decreases, and the activation of a metallic surface takes place. The higher affinity for a metal an anion has, the more strongly the screening effect and activation of a metallic surface occur.

The experimental data given in [69,70] prove the activation of a metallic surface during the chemosorption of fluorine ions on it. It was established that the activation of a metallic surface results in the formation of only superoxides during their further high-temperature air oxidation. Particularly, on a surface of Zr and Zr-2.5% Nb alloy, mainly ZrO_2_+δ forms over a wide temperature range (600–1000 °C) (δ—excess of oxygen from its stoichiometric content in zirconium dioxide). The formation of a brittle phase of oxygen solid solution in α-Zr almost does not exist.

It should be noted that after the high-temperature (T > 1300 K and P_O2_ ≥ 10^4^ Pa) treatment of Zr in an oxygen-containing gaseous atmosphere, the p-n junction exists in the forming oxide layer [76]. The original version of these results can be seen in supplement material.

**Figure 4:**
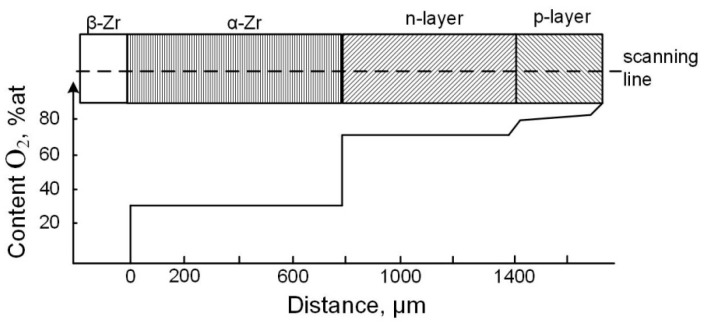
Arrangement of layers in “Zr-oxide layer” system and the oxygen content changing through their thickness. The coating was synthesized under the high-temperature (1570 K) treatment of Zr for 1 h (P_O2_ ≈ 21280 Pa) [76]. Reprinted with permission from Ref. [76]. 1994, Moscow: MISiS.

This coating has two layers: an inner black one with a lack of oxygen from its stoichiometric content in zirconium dioxide (n-conducting layer), and an outer p-conducting layer of greyish-white color (Figure 4).

**Figure 5:**
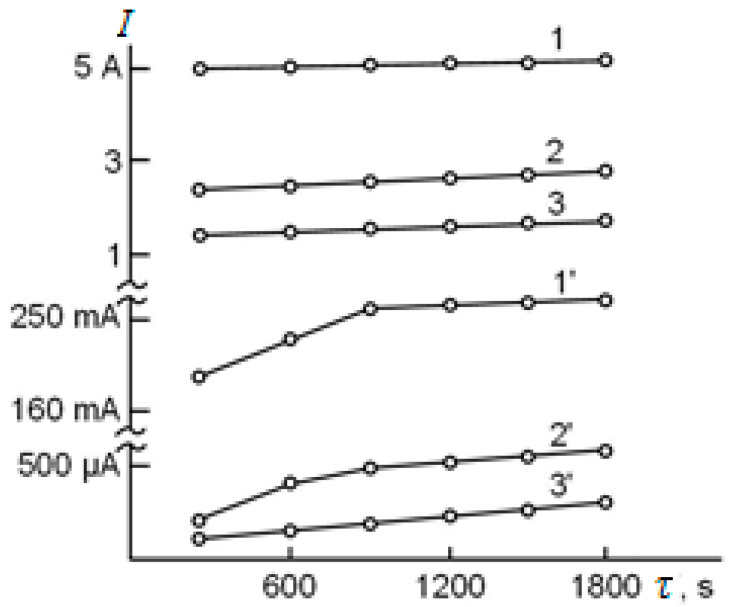
Evolution of reverse (1, 2, 3) and direct (1′, 2′, 3′) current as function of oxidation under P_O2_ ≈ 21,280 Pa, T = 1570 K and E_external_: 1, 1′–5 V; 2, 2′–2 V; 3, 3′–1 V [76]. Reprinted with permission from Ref. [76]. 1994, Moscow: MISiS.

There is a charged bilayer at the p-n junction interface. With a direct connection of an external source to this junction (a negative pole is connected with a metal, the positive one with an oxide layer) under voltage in the E_external_ range of 1–5 V, high temperature and oxygen partial pressures, the measured current values are not high due to high resistance of the outer layer. However, a reverse connection to the p-n junction results in an increase in the current by several orders (Figure 5). This connection of an external supply source of current leads to the breaking of the p-n junction. Asymmetry of electric conductivity during direct and reverse passing of the current through “Zr-oxide layer” system does not occur if a coating grown under the oxygen partial pressure in a gaseous atmosphere consists of only ZrO_2_–δ (n-conducting layer) [76].

This experimental data confirms that Zr and its alloys are so-called “valve” metallic materials. Valve materials are capable of electrochemically forming adhesive strong surface oxide layers with unipolar conductivity in the material–oxide–electrolyte system. Recently, plasma electrolytic modification (PEM) and microarc oxidation (MAO) are becoming increasingly popular for modifying the surface of valve materials [1,11,27,28,29,30]. The PEM method gives possibilities to synthesize nanostructured ceramic-like layers on the surface that have high adhesion strength, good corrosion-protective ability and wear resistance. After this way of treatment, materials acquire fundamentally new properties; in particular, their corrosion resistance becomes several orders of magnitude higher than that of untreated alloys. Wear resistance also increases many times.

As noted above, zirconium fully belongs to the class of valve materials. Below is a review of some of the results obtained for the PEM modification of Zr and its alloys.

## 5. Formation of Oxide-Ceramic Layers on the Surface of Zr and Its Alloys by PEM

On the alloy Zr-2.5 wt.% Nb, which is applied for pressure tubes of nuclear reactors, oxide coatings were synthesized using plasma electrolytic modification (PEM) [77,78]. The effect of treatment factors such as electrolyte composition and current density on microstructure and coating properties was systematically investigated. Potentiodynamic polarization corrosion and wear tests were performed, and the obtained results were compared with commercial autoclaved black oxide coating. The main treatment modes used by the authors are shown in Table 3.

**Figure 6:**
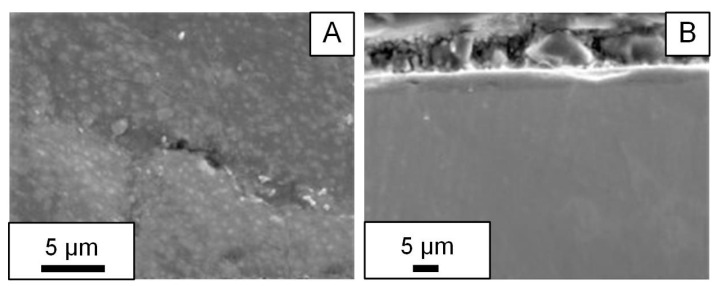
SEM images of the morphology (**A**) and cross-section (**B**) for S1 sample treated in electrolyte I (Table 3) [77]. Reprinted with permission from Ref. [77]. 2010, WIT Press.

Sample S1 was modified at a current density of 0.05 A/cm^2^ and exhibited a relatively better dense and continuous oxide film (see Figure 6). Samples fabricated at a higher current density exhibit significant porosity. The explanation by the authors is that at higher current densities, the discharges are more intense, and pores are a consequence of discharge channels.

Potentiodynamic polarization tests were carried out at room temperature (20 °C) in a 4.8 wt.% (0.2 mol/L or 1400 ppm Li^+^) LiOH solution which has a very strong corrosive effect on Zr alloys using a Bio-Logic SP-150 potentiostat/galvanostat. The exposure area of the samples was 1 cm^2^. Scans were conducted from −0.5 V to 2 V. The results are shown in Table 4.

It can be seen that all samples coated by PEM had much lower (as much as one to two orders of magnitude) i_corr_ compared to untreated Zr-2.5Nb substrate, and the E_corr_ values (−0.21 V to −0.28 V) are higher than that of the substrate (−0.35 V). From the data in Table 4, it can be seen that PEM coatings obtained in electrolyte I have a better corrosion resistance than those made in electrolyte II. This is in agreement with previous studies of the production of PEM coatings on Al, where alkaline electrolytes are widely used, and too high a pH value may have a negative effect on the growth of passivating films [79].

**Figure 7:**
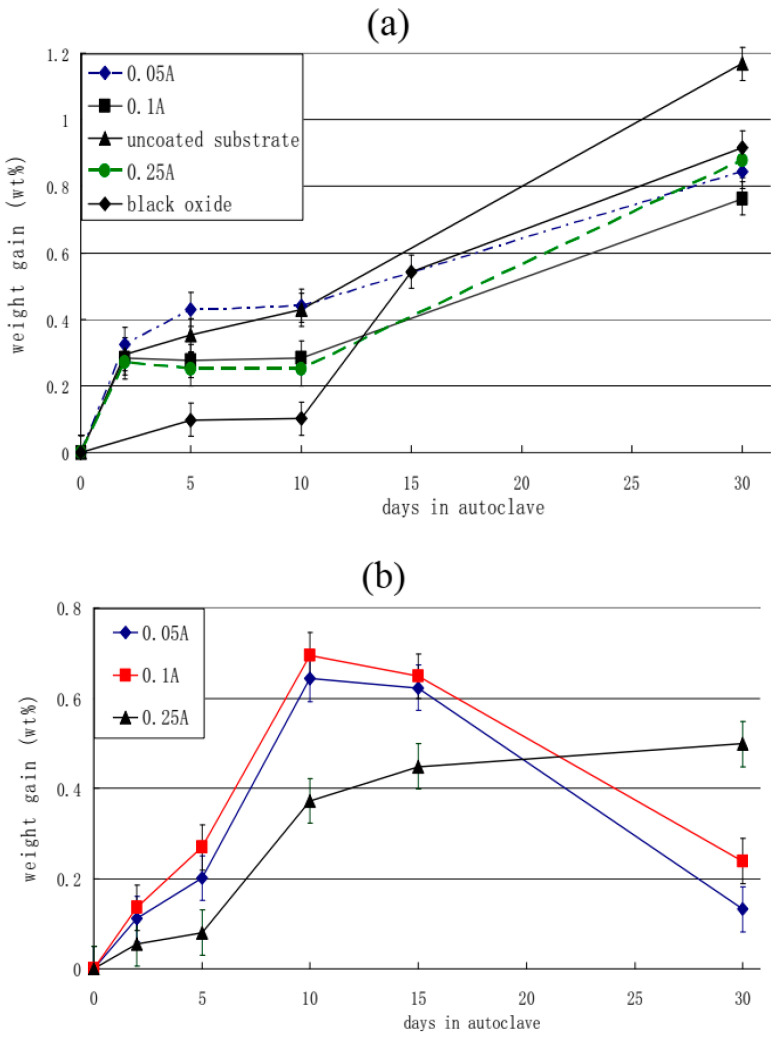
Weight gain vs. treatment time: (**a**) in electrolyte I, substrate and black oxide coating; (**b**) in electrolyte II (see Table 3) [77,78]. Reprinted with permission from Ref. [78]. 2010, University of Windsor.

The authors of [77] performed gravimetric studies of the modified alloy’s ability to resist oxidation. Figure 7 shows the weight gain changing with the exposure time in the autoclave for up to 30 days at 300 °C and the high pressure of 10 MPa in 0.05 mol/L LiOH solution. The weight of PEM coatings increased rapidly in the first two days, and then growth slowed down. This phenomenon is explained by the researchers as the penetration of oxygen from the solution into the insufficiently dense outer layer of the PEM coating and the subsequent formation of ZrO_2_ along with other intermediate lower oxides. It has been also noted that near the oxide/metal interface, there is a very thin inner dense layer which serves as an excellent barrier to oxygen penetration and explains the reduction in corrosion rate during two days of exposure. However, after reaching a certain point (called a “transition point” by the authors), all coated samples demonstrate a sharp increase in the corrosion rate. Comparing the two groups (Table 3), it can be seen that Group A samples treated with electrolyte I had a longer pre-transition period (ranging from 10 to 30 days) than Group B samples treated with electrolyte II (ranging from 5 to 10 days). Group A weight gain was almost the same (0.76–0.87%). However, due to the destruction of oxide layers in Group B samples, it was difficult for the authors to measure the change in weight after 10 days of exposure in the autoclave; nevertheless, data on the increase in weight in Group B after the 10-day mark show a rate decrease compared to the shorter exposure time.

The uncoated substrate and black oxide-coated samples were also tested under exposure for up to 30 days. As with PEM-coated samples, the weight of the substrate initially increases rapidly due to the formation of a protective film. Unlike Group B, the PEM coatings treated with electrolyte II, where the coatings have been completely removed, the coating formed on the native substrate consists of several thin layers formed parallel to the oxide/metal interface. When a certain oxide thickness is reached, the outer coating layer is cracked and separated from the sample surface. In this case, the weight gain value shown in Figure 7a for the substrate is much lower than its actual value. However, the final mass gain (about 1.18%) is still higher than for the PEM coating. The black oxide coating has excellent corrosion prevention properties in the first 10 days (better than all PEM coatings). However, over a long time of exposure, it showed the same weight gain as PEM coatings (0.92%).

The tribological properties were tested by a pin-on-disc tribometer at room temperature. Rotating mode with a sliding speed of 0.1 m/s was used for both the substrate and the PEM samples. All of the PEM coatings were tested under dry conditions at 2 N normal load and 1000 m sliding distance. The uncoated substrate was tested at the same parameters but only to a 50 m sliding distance. The PEM coatings (S1) showed the best wear resistance. The wear test results are summarized in Table 5. The main conclusion is that almost none of the PEM coatings failed after a 1000 m sliding distance during testing.

Investigations into the effects of process parameters (both electrolytic and electrical) on PEM coating formation for Zr-2.5Nb alloy and on the corrosion and wear properties of the coatings showed the following:

- The best coatings from the perspective of density and continuity were produced at higher Na_2_SiO_3_+KOH ratios (10:1 cf 1:1, Table 3), i.e., lower pH values; lower current densities; and longer treatment duration.
- PEM coatings improved the corrosion resistance of the Zr-2.5Nb substrate (the polarization resistance (R_p_) was one to two orders of magnitude higher than that of the substrate).
- PEM coatings of 5 μm thickness have a better wear resistance than the commercial autoclaved black oxide coating under both dry and water-lubricated conditions.
- PEM coatings obtained in a 10:1 Na_2_SiO_3_+KOH electrolyte exhibit much better corrosion resistance and lower weight gain than the Zr-2.5Nb substrates after 30 days in an autoclave (exposure at 300 °C, high pressure 10 MPa in 0.05 mol/L LiOH solution). The above-mentioned black oxide coating, having a very low weight gain value in the first 10 days, by 30 days demonstrates a final weight gain very close to that of the PEM samples made at an electrolyte component ratio of 10:1.

In [46], the modes of formation are described and the characterization of the PEM coating on Zr alloy of grade 702 is given. The resulting composite material (metal zirconium base–oxide-ceramic outer layer) was proposed to be used in the creation of a heat-decoupling element between the hood and the frame of the main mirror of the T-170M telescope of the International Space Observatory “Spectrum-UV”. The modes of formation of PEM coatings and the data for determining their properties are given in Table 6.

The table shows the voltage (U) and duration of PEM (τ) in acid and phosphate electrolytes. The thickness of the coatings (h), the thermal conductivity of the composite material (λ_CM_) and the maximum specific weight of easily condensable substances as an indicator of gas evolution (M) were determined by instrumental methods. The total porosity of the coatings (P_g_) and the average nominal pore diameter (d) were calculated geometrically from metallographic images of cross-sections. The thermal conductivity of the coating (λ_coat_) was calculated from the difference between the thermal conductivities of the alloy (22.7 W/m·K) and the composite material (λ_CM_), taking into account their thicknesses for each of the samples.

Because of the study, it was found that a composite material consisting of zirconium and a PEM coating applied to it has a low thermal conductivity (less than 2 W/m K). It is shown that with an increase in the thickness of the PEM coating, its porosity decreases, while the average nominal pore diameter increases, which leads to an increase in the thermal conductivity of the PEM coating and makes the use of thick PEM coatings inappropriate. In addition, the gas evolution of the resulting composite material is below critical, which is considered to be the maximum for easily condensable substances 5·10^−4^ g/cm^2^. This makes it possible to use these materials in open space conditions. At the same time, the material treated in two electrolytes—phosphate and acid—has the lowest gas evolution.

In [80], the effect of the electrolyte composition on the protective properties of a PEM coating on an E110 Zr-1%Nb zirconium alloy is discussed. When evaluating the operational properties of the coatings to identify the most effective electrolyte, the topography and microstructure of the coatings, the elemental and phase compositions were investigated, and electrochemical and accelerated operational tests were carried out.

**Figure 8:**
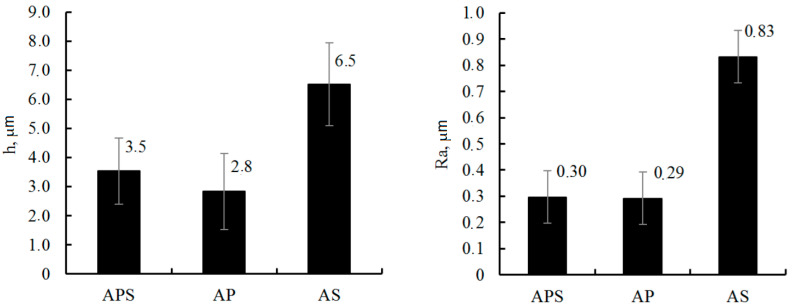
Thickness and roughness of PEM coatings obtained in various electrolytes [80]. Reprinted with permission from Ref. [80]. 2019, the authors.

Plasma electrolytic oxidation was carried out at a constant temperature of 20 ± 1 °C for 10 min in a pulsed unipolar voltage with a pulse frequency of 500 Hz. The duty cycle of the pulses was 50%, and the constant current density was 10 A/dm^2^. The following aqueous electrolytes were used: 1 g/L KOH + 2 g/L Na_2_SiO_3_ + 2 g/L Na_4_P_2_O_7_ (APS), 1 g/L KOH + 2 g/L Na_4_P_2_O_7_ (AP) and 1 g/L KOH + 2 g/L Na_2_SiO_3_ (AS). The obtained coatings with a thickness of 2.8 to 6.5 µm with a surface roughness Ra of 0.29 to 0.74 µm had a characteristic porous structure, which is associated with the effect of micro-discharges during processing. The maximum pore size reaches 10 µm, and small pores have a size of 2–3 µm. The highest porosity of the coating, P = 20%, is observed for the sample treated in AS, and the smallest, P = 3%, is achieved by treatment in the APS electrolyte. The thickness and roughness of the coating during the treatment of samples in the AS electrolyte are greater than those during processing in APS and AP electrolytes, where these parameters have lower values and are almost identical (Figure 8).

X-ray phase analysis showed that the coating contains crystalline phases of monoclinic *m*-ZrO_2_ and tetragonal *t*-ZrO_2_ modifications of ZrO_2_. No high-temperature cubic phase was found. The monoclinic phase of *m*-ZrO_2_ is predominant during treatment in all electrolytes. The high-temperature tetragonal *t*-ZrO_2_ modification is found in the coatings obtained in APS and AS electrolytes at the level of 2%; in the AP electrolyte, it is found only at the trace level.

The results of evaluating the adhesion strength of the coatings, carried out by the destructive method on the device “Revetest, CSM instruments, Switzerland” equipped with a diamond indenter with a radius of curvature of 200 microns, are presented in Table 7.

The highest value of the critical load at which the PEM coating is destroyed was recorded for a sample treated in APS electrolyte. The sample treated in AS electrolyte had the lowest adhesion strength, which can be attributed to the high porosity and surface roughness.

**Figure 9:**
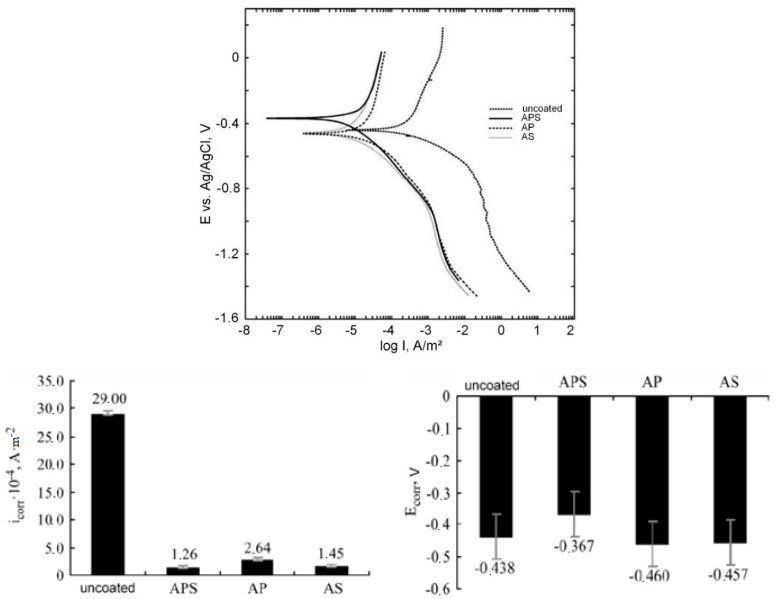
Polarization curves, potential and corrosion current for samples treated in various electrolytes [80]. Reprinted with permission from Ref. [80]. 2019, the authors.

Electrochemical studies were carried out using an Elins R-5X potentiostat (Russia) with a 0.1 M LiOH solution in a three-electrode cell with a silver chloride reference electrode and a platinum counter electrode. The measurement of the electrode potential was carried out for 2 h to reach a steady-state value. The measurement range of the polarization curves was from −1.5 to +0.2 V (relative to the reference electrode), and the scanning speed was 0.25 mV/s. The corrosion potential and current were calculated by the Tafel method from the polarization curves. The results of electrochemical corrosion studies are shown in Figure 9. It can be seen that the value of the free corrosion potential is greatly influenced by the composition of the electrolyte. The value of the potential for free corrosion indicates the potential of the surface of the samples to actively participate in the chemical reaction. The coating obtained in APS electrolyte has a more passive surface in contrast to the untreated sample and samples processed in other electrolytes. This is evidenced by the large value of E_corr_. The E_corr_ value behaves according to the ratio of the atomic percentages of zirconium and oxygen in the PEM coating. An increase in the proportion of the substrate material (zirconium) in the coating leads to an increase in surface activity. Therefore, treatment in AP and AS electrolytes makes the surface more active, which does not improve the protective characteristics.

The rate of the chemical corrosion process is determined by the value of the corrosion current i_corr_. The values of corrosion currents in the treated samples are more than an order of magnitude lower than the value in the untreated sample. The lowest corrosion current is observed for the sample treated in APS electrolyte. The nature of the change in the corrosion current depending on the composition of the electrolyte is also associated with a change in the ratio of atomic percentages of zirconium and oxygen in the PEM coating.

**Figure 10:**
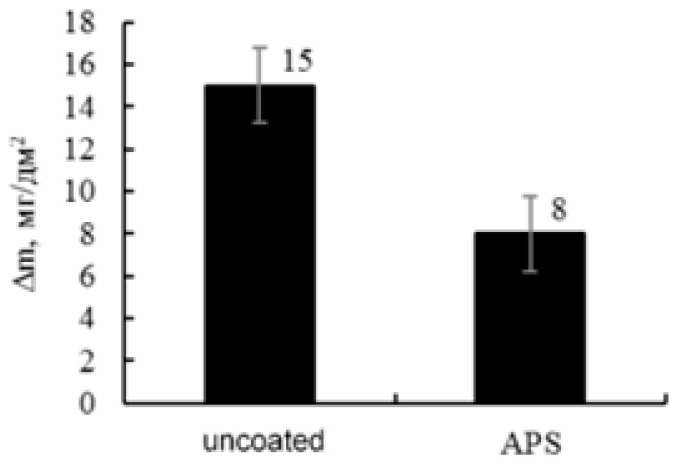
Weight gain after accelerated testing [80]. Reprinted with permission from Ref. [80]. 2019, the authors.

Taking together all the studies carried out by [80], the most effective electrolyte (among alkaline silicate–phosphate electrolytes) for the PEM of the zirconium alloy Zr-1% Nb is the APS electrolyte. Samples processed in this electrolyte and uncoated samples were subjected to accelerated performance testing. Testing was carried out in a 250 mL Midiclave autoclave at 400 °C in steam for 70 h. Samples before and after testing were weighed on an analytical balance with an accuracy of 0.1 mg. The results are shown in Figure 10.

It can be seen that the weight index of corrosion—weight gain—is higher for the untreated sample. A dark dense matte film was formed on the surface of the uncoated sample; the presence of a white film was noted on the coated samples. At identical phase compositions of the corrosion products, this may be due to the effect of amorphous Si, Na and K compounds fixed in the coating. After accelerated performance tests for aqueous corrosion of the PEM-coated specimens, X-ray analysis did not detect the presence of *t*-ZrO_2_; only the monoclinic crystalline phase was recorded. Apparently, at elevated temperatures, a gradual transition of zirconium oxide to a thermodynamically more equilibrium state occurs.

Finally, the authors of [80] concluded that the coatings obtained in a silicate–phosphate electrolyte have the best protective properties.

A continuous ceramic coating of 8 μm thick on Zr-1%Nb alloy was formed by plasma electrolytic oxidation in an alkaline phosphate electrolyte [81]. The high-temperature steam oxidation behavior of initial and PEM-coated alloys was evaluated in a water vapor environment at 900–1200 °C using a thermogravimetric analyzer. Morphologies, compositions and constituents of phases before and after the steam impact were characterized. It was found that the mass gain of PEM-coated specimens was 38%, 56%, 93% and 100% compared with untreated Zr alloy after 3600 s steam oxidation at 900 °C, 1000 °C, 1100 °C and 1200 °C, respectively. This means that the steam oxidation resistance below 1000 °C for the mentioned alloy obviously grew due to PEM and barely improved at higher temperatures. The authors connect the mass gain rate in high-temperature steam tests with the oxygen diffusion coefficients in the β-Zr, α-Zr(O) and ZrO_2_ phases. In addition, it was shown that during steam oxidation at 900–1200 °C, up to 1100 °C the oxide layers for initial and PEM-coated Zr-1%Nb alloy contain monoclinic zirconia (*m*-ZrO_2_) and a small amount of tetragonal zirconia (*t*-ZrO_2_) phases, but the *t*-ZrO_2_ phase disappears at 1200 °C.

**Figure 11:**
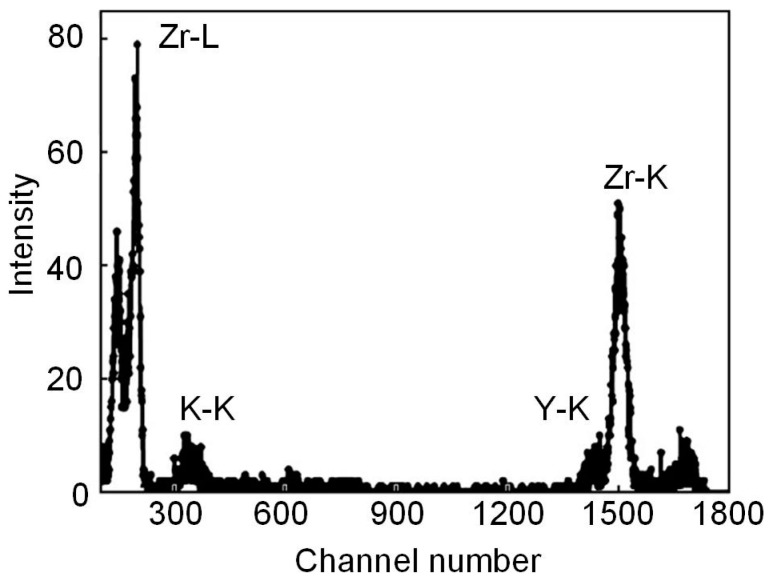
Spectrum of characteristic X-ray emission excited by protons with the energy of 1.5 MeV in a sample with PEM coat formed on zirconium in the electrolyte containing Y_2_O_3_ nanopowder [82]. Reprinted with permission from Ref. [82]. 2012, Pleiades Publishing, Ltd.

Oxide-ceramic PEM coatings were synthesized during microarc oxidation of commercially pure zirconium in an electrolyte containing 6 g/L NaAlO_2_, 2 g/L KOH and 1 g/L sodium hydroxide water glass at room temperature without and with the addition of Y_2_O_3_ nanopowder at the rate of 2 and 5 g/L [82]. To study the incorporation of yttrium oxide Y_2_O_3_ into the coating structure, the method of proton-induced X-ray emission (PIXE) was used. The measurement of the elemental composition of materials using the PIXE method is based on the registration of X-ray radiation excited in a sample by accelerated protons with a resolution of 180 eV at an energy of 5.9 keV by a Si-PIN diode. The detector was installed at an angle of 135 ° to the direction of the proton beam-probe, creating a solid angle of the detector Ω = 1.5·10^−5^ sr. The beam was monitored by the output of backscattered protons recorded at an angle of 160° to the direction of the proton beam-probe. The diameter of the proton probe was 1 mm, and the beam current was tens of nanoamperes. The time of one measurement cycle did not exceed 10 min. In the spectrum of characteristic X-ray radiation (Figure 11), along with the K- and L-lines of zirconium, the emission peak of the K-line of yttrium atoms is clearly observed. When measuring this spectrum, a 10 μm thick aluminum foil filter was installed in front of the X-ray counter window to reduce the overload of the detector by low-energy photons.

The studies carried out made it possible to establish the atomic concentration of yttrium relative to zirconium in the PEM coating. Thus, after microarc oxidation of zirconium in an electrolyte with the Y_2_O_3_ nanopowder concentration of 2 g/L, the amount of Y relative to Zr was 3.5%. An increase in the concentration of the Y_2_O_3_ additive in the electrolyte to 5 g/L almost proportionally increases the atomic concentration of Y relative to Zr in the PEM coating to 8.3%. It is known that the Y_2_O_3_ additive is used in the production of cubic zirconia (fianit) as a stabilizer for the cubic modification of ZrO_2_. On this basis, the authors of [82] assumed that the incorporation of yttrium oxide nanopowder into an oxide-ceramic PEM coating is capable of stabilizing the high-temperature tetragonal and cubic modifications of zirconium dioxide.

In [19], PEM coatings were obtained on the surface of tubes 8 mm in diameter made from zirconium alloy grade E110 (Zr + 1% Nb) in electrolytes of various compositions with additions (up to 5 g/L) of yttrium oxide and aluminum oxide nanopowders. The PEM process was carried out in the anodic–cathodic mode at Ia/Ic = 1 with a total density of the anodic and cathodic currents of 10 A/dm^2^. The duration of the PEM treatment was 60 min.

To study the elemental composition of PEM coatings, the method of nuclear backscattering spectrometry (NBS) of 7.7 MeV protons was used [20]. For the monitoring by the proton beam, tantalum foil was applied to shield the surface of the sample under study. The morphology of PEM coatings was studied using a Quanta-600 scanning electron microscope (SEM) with attached equipment for elemental analysis. X-ray phase analysis was performed on a DRON-4 diffractometer in Cu Kα radiation.

**Figure 12:**
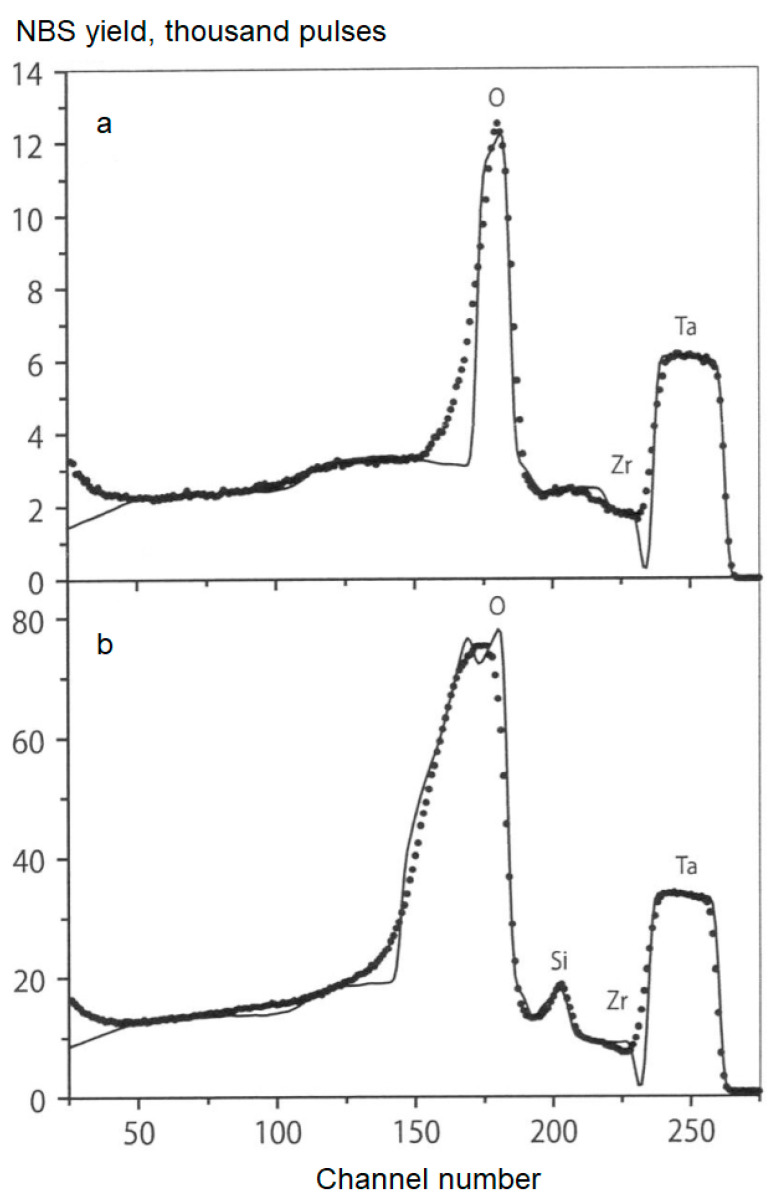
NBS spectra of the surface layer for the E110 zirconium alloy after PEM treatment in an electrolyte containing (**a**) 2 g/L KOH and (**b**) 2 g/L KOH + 12.5 g/L Na_2_SiO_3_ [19]. Reprinted with permission from Ref. [19]. 2012, Moscow: MISiS.

The NBS spectra shown in Figure 12 represent a superposition of energy-separated spectra of protons scattered on Ta foil (230–265 channels) and the studied PEM coating (channel numbers Nk < 230). Information about PEM coating composition was obtained by modeling the spectra using the NBS program and comparing the calculated and experimental data as described in [83]. Thus, in the case of PEM treatment in an alkaline electrolyte containing 2 g/L KOH (Figure 12a), it was found that the oxide coating consisting of zirconium dioxide ZrO_2_ has a thickness of 7 μm. The addition of 12.5 g/L Na_2_SiO_3_ to this electrolyte (Figure 12b) leads to the formation of a two-layer coating consisting of an outer layer (a mixture of zirconium and silicon oxides in a ratio of 4:1, ~5 μm thick) and an inner ZrO_2_ layer 12 μm thick.

The addition of 2 g/L of Al_2_O_3_ nanopowder to the alkaline electrolyte does not lead to a noticeable change in the thickness and composition of the PEM coating. Increasing the concentration of this component to 5 g/L leads to a 2-fold increase in the coating thickness (from 7 to 14 μm), but also without changing the composition. At the same time, the addition of 5 g/L of Al_2_O_3_ nanopowder to the silicate alkaline electrolyte (12.5 g/L Na_2_SiO_3_ + 2 g/L KOH) does not affect the thickness or the composition of the coating. PEM coatings obtained in an aluminate–silicate alkaline electrolyte (6 g/L NaAlO_2_ + 2 g/L Na_2_SiO_3_ + 2 g/L KOH) with and without additions of 2 and 5 g/L of Y_2_O_3_ nanopowder turned out to be practically the same thickness, ~20 μm.

**Figure 13:**
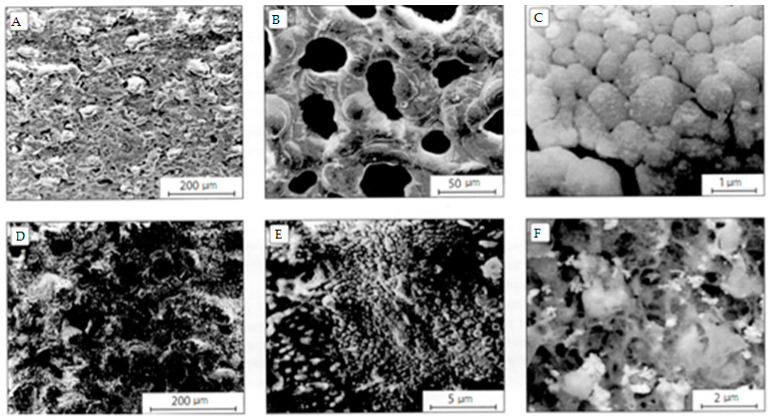
Surface morphology of PEM coatings on zirconium alloy E110: (**A**) initial sample; (**B**) PEM treatment in an alkaline electrolyte; (**C**) the same +5 g/L of Al_2_O_3_ nanopowder; (**D**) PEM treatment in an aluminate–silicate alkaline electrolyte; (**E,F**) the same +2 g/L of Y_2_O_3_ nanopowder [19]. Reprinted with permission from Ref. [19]. 2012, Moscow: MISiS.

SEM studies of the initial sample surface revealed traces of mechanical treatment of the E110 zirconium alloy (Figure 13A). After PEM treatment in an alkaline electrolyte, traces of mechanical treatment disappeared and a surface morphology typical of PEM coatings was observed: a porous structure with a pore size of up to 10 μm (Figure 13B). The addition of 2 g/L of Al_2_O_3_ nanopowder to this electrolyte practically did not change the picture. Almost no precipitation of Al_2_O_3_ nanopowder was observed on the coating surface, but elemental analysis showed that the PEM coating contains aluminum (~0.6 at.%). The PEM coating formed in the same electrolyte, but with the addition of 5 g/L of Al_2_O_3_ nanopowder (Figure 13C), has larger pore morphology with a pore size of ~50 μm.

At higher magnification, the structure of the coating material is visible—grains ranging in size from 50 nm to 2 μm with intergranular cracks (Figure 13D). Separate conglomerates of Al_2_O_3_ nanopowder are found on the surface, and elemental analysis shows the Al content in the coating in the amount of 2–2.5 at.%. The surface of the PEM coating obtained in an aluminate–silicate alkaline electrolyte without (Figure 13E) and with additions of 2 and 5 g/L of Y_2_O_3_ nanopowder (Figure 13F,G) is inhomogeneous, has a developed relief and has a significant roughness. It contains small particles and pores, as well as areas with a dendritic structure, typical for the electrochemical formation of oxide (Figure 13F). At higher magnification, it can be seen that when the content of Y_2_O_3_ nanoparticles in the aluminate–silicate alkaline electrolyte is 5 g/L, they partially coagulate and form clusters (Figure 13H).

The results of X-ray phase analysis showed that PEM coatings formed in alkaline and silicate alkaline electrolytes without and with additions of Al_2_O_3_ nanopowder (2 and 5 g/L) are characterized by the monoclinic phase of zirconium dioxide *m*-ZrO_2_. A change in the texture of the zirconium under the coating was also found. After PEM treatment in an aluminate–silicate alkaline electrolyte, in addition to *m*-ZrO_2_, the tetragonal phase of zirconium dioxide *t*-ZrO_2_ (in the same amount) appears in the coating, apparently due to the creation of sufficient temperature conditions for this. After adding 2 g/L of yttrium oxide nanopowder to this electrolyte, the *t*-ZrO_2_:*m*-ZrO_2_ ratio becomes 4: 1. With an increase in the concentration of Y_2_O_3_ nanopowder to 5 g/L, the X-ray diffraction pattern shows reflections only for *t*-ZrO_2_. With a further increase in the concentration of Y_2_O_3_ nanopowder to 7 and 10 g/L, only the cubic phase of zirconium dioxide *c*-ZrO_2_ is observed in the surface layer of the PEM coating, and after its partial grinding, traces of *m*-ZrO_2_ are also noticeable. Thus, X-ray phase analysis confirms the possibility of stabilization of high-temperature modifications of zirconium dioxide *t*-ZrO_2_ and *c*-ZrO_2_ during PEM treatment of zirconium alloys in electrolytes with additives of yttrium oxide nanopowder [19].

Stabilizing additives can be added to the coating composition not only from the slurry electrolytes. In [84], oxide coatings were produced on pure zirconium by the microarc oxidation method in the electrolytes containing sodium silicate and different amounts of yttrium acetate tetrahydrate (YAT). The electrolytes with four different compositions were prepared by using a certain amount of sodium silicate and a variable amount of YAT by dissolving them in distilled water as given in Table 8. The PEM coating was carried out by employing a unique self-made asymmetric AC power supply. The processing duration for each sample was 60 min.

**Figure 14:**
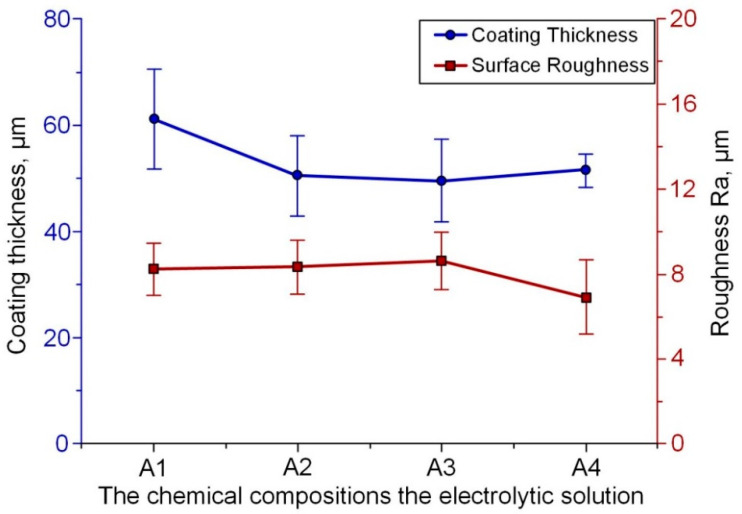
The change in coating thickness and surface roughness (Ra) for PEM coating on Zr in A1, A2, A3 and A4 electrolytes (the compositions are indicated in Table 8) [84]. Reprinted with permission from Ref. [84]. 2014, Proceedings of the 4th International Congress APMAS2014.

The obtained average coating thickness was approximately 50 µm (30–65 µm range) and slightly depended on the amount of YAT in the electrolyte. The average surface roughness changed from 6.85 µm to 8.23 µm depending on the composition of the electrolytes. The surface roughness of the untreated zirconium substrates just before coating was approximately 0.16 µm (see Figure 14).

**Figure 15:**
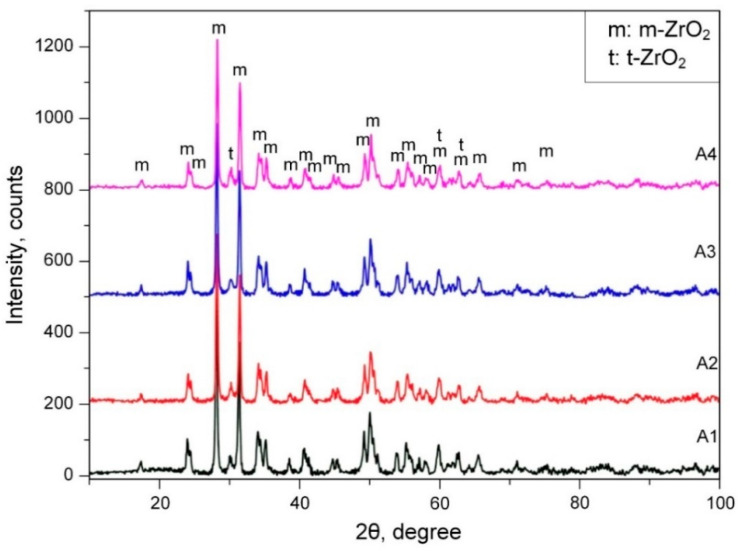
The XRD patterns of PEM coating formed on zirconium substrates in A1, A2, A3 and A4 electrolytes [84]. Reprinted with permission from Ref. [84]. 2014, Proceedings of the 4th International Congress APMAS2014.

The X-ray diffraction (XRD) analysis data showed the presence of the *t*-ZrO_2_ high-temperature phase (Figure 15). This high-temperature phase is usually stabilized at room temperature by oxides such as MgO, Y_2_O_3_ and CaO [61,62,63,64,65,66]. The presented data also evidence that when the temperatures of metal and metal oxide sharply decrease during the PEM process, ZrO_2_ phases can be stabilized in various crystal structures because of non-equilibrium transformation due to the rapid cooling effect (quenching) of the cold electrolyte. As seen from the XRD patterns, all of the coatings consist of *m*-ZrO_2_ and *t*-ZrO_2_ phases. The presence of the tetragonal phase of ZrO_2_ was explained by authors of [84] as due to the partial stabilization effect of *t*-ZrO_2_ because of non-equilibrium transformation.

**Figure 16:**
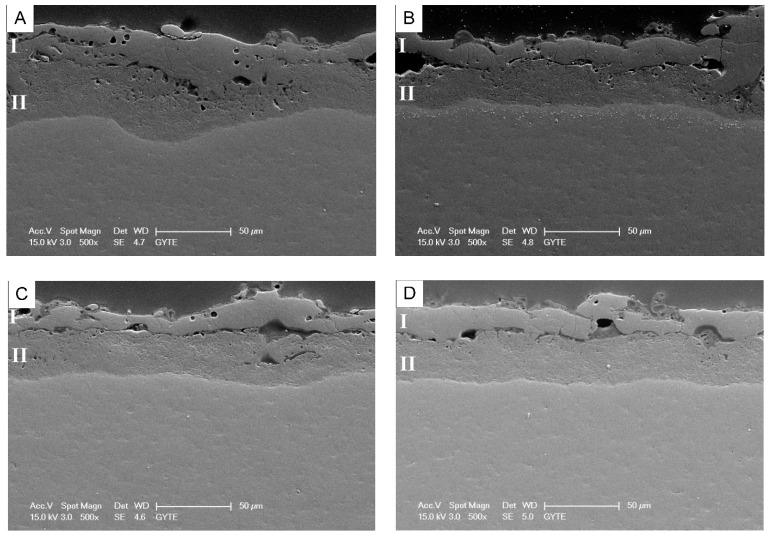
The SEM images of cross-section PEM-coated Zr in different electrolytes. (**A**) A1, (**B**) A2, (**C**) A3 and (**D**) A4 (Table 8) [84]. Reprinted with permission from Ref. [84]. 2014, Proceedings of the 4th International Congress APMAS2014.

It was also described that the structure of the obtained PEM coatings has two apparent parts: the outer dense area (I) with some defects (cracks and small pores) and the porous inner layer (II). The distinction between areas I and II becomes more significant with the addition of YAT into the electrolyte (Figure 16).

The patent [85] proposes a method of forming heat-shielding coatings on gas turbine blades for aircraft engines and power plants made of nickel or cobalt alloys. The method includes a heat-resistant sublayer deposition and the formation of a ceramic layer of zirconium dioxide stabilized with yttrium oxide on the sublayer. In this case, the heat-resistant layer is formed by the ion-plasma method with a thickness of 10 µm to 30 µm, whereupon a layer with a thickness of 30 µm to 500 µm from the alloy of zirconium with yttrium (the content of yttrium is 5–9 wt.%) is applied. Then the deposited layers are subjected to microarc oxidation: a layer of zirconium with yttrium for the entire thickness, and a heat-resistant layer for a thickness of 1 μm to 3 μm. Despite the fact that the heat-resistant sublayer has a composition (wt.%) of Cr, from 18% to 34%; Al, from 3% to 16%; Y, from 0.2% to 0.7%; and Ni, the rest or Cr, from 18% to 34%; Al, from 3% to 16%; Y, from 0.2% to 0.7%; Co, from 16% to 30%; and Ni, the rest, patent owners testify on the possibility of its microarc oxidation in an environment of 3–5% aqueous solution of ammonium phosphate when a positive potential from 300 to 1100 V is supplied to the part to be coated. It is stated that the resulting heat-shielding coatings have high performance characteristics and increased adhesive strength.

No less interesting is the method of forming a ceramic coating based on zirconium dioxide on articles made of titanium alloys [86]. The technology of its creation includes the initial electro-plasma spraying of dioxide followed by modification by conducting microarc oxidation in an aqueous alkaline electrolyte based on sodium hydroxide with a concentration of 1–3 g/L in the anode mode at a constant current density (2–2.5)·10^3^ A/m^2^ lasting 20–30 min.

Our research group studied the structure and composition of PEM coatings synthesized on the Zr-1% Nb alloy [87]. In slurry electrolytes containing 9 g/L of sodium silicate, 5 g/L of sodium hypophosphite and 4–6 g/L of yttria nanopowder additive, surface layers up to 120 μm thick were created. The AC mode at total current densities of 20–30 A/dm^2^ was used, and processing time was 30–60 min.

The coating thickness after 30 min of treatment in an electrolyte with a concentration of yttria nanopowder additive of 4 g/L at the current density of 20 A/dm^2^ was ~30 μm, and after 60 min, it was ~80 μm. With an increase in the nanopowder concentration up to 6 g/L, the coating thickness grows to ~100 μm. After an increase in current density up to 30 A/dm^2^, the thickness reached ~120 microns. At the same time, it was noticed that upon modification at a higher current density, the surface of the coating became rougher.

**Figure 17:**
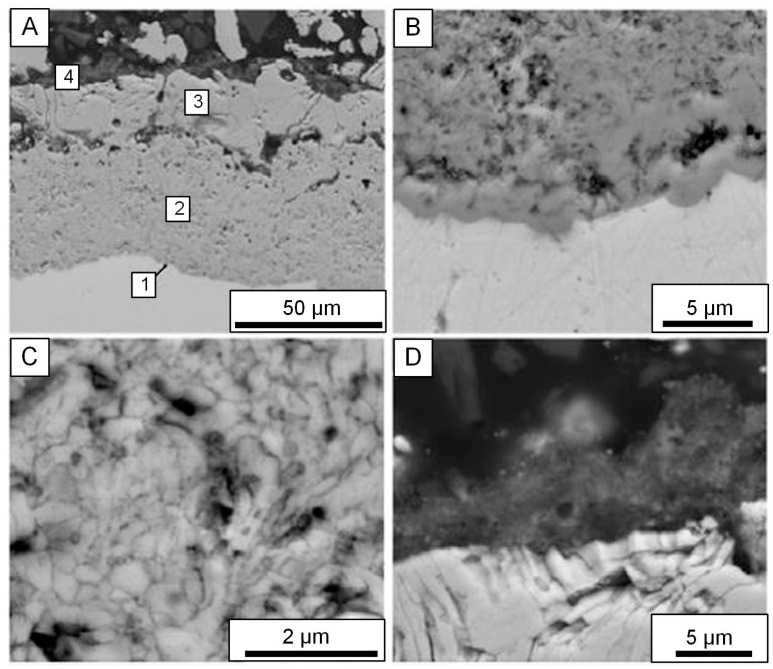
The BSE images of the coating cross-section which was formed on the Zr-1% Nb alloy at current density 20 A/dm^2^ for 60 min in the slurry electrolyte with 4 g/L Y_2_O_3_ nanopowder. (**A**) Barrier (1), intermediate (2), outer (3) and superficial (4) layers; (**B**) barrier layer (1) at the interface with the alloy; (**C**) the crystallites in the layer (2); (**D**) the superficial layer (4) with a high content of silicon oxides and aggregated Y_2_O_3_ nanoparticles [87]. Reprinted with permission from Ref. [87]. 2016, Elsevier B.V.

Our analysis of the coating cross-section obtained on the Zr-1% Nb alloy in the slurry electrolyte with 4 g/L of Y_2_O_3_ nanopowder is illustrated by SEM images in Figure 17A. It is visible that the coating consists of four parts: (1) barrier, (2) intermediate, (3) outer and (4) superficial layer which contains conglomerates of yttria nanoparticles.

The barrier layer, less than 1 μm thick, is rather dense, but its thin discharge channels are traced (Figure 17B). The intermediate layer is the thickest and consists of heteraxial crystallites with dimensions of 0.2–0.3 μm and pores with sizes of about 0.2 μm (Figure 17C). It should be noted that the similar structure promotes a decrease in heat conductivity of PEM coatings on zirconium alloys. Large pores about 5 μm in size and also through or deadlock discharge channels characterize the outer layer about 25 μm thick which is designated as (3) in Figure 17A. The superficial layer about 5 μm thick is enriched with electrolyte components and contains aggregated Y_2_O_3_ nanoparticles (Figure 17D).

EDX microanalysis of the cross-section for the sample treated for 60 min in the electrolyte comprising 4 g/L yttria nanopowder (Figure 17A) showed an increase in yttrium concentration from the barrier layer up to the superficial layer. The intermediate part of PEM layer (2) contains about 2.5 at.% Y, 30 at.% Zr, 65 at.% O and 2 at.% Si. The outer layer (3) contains about 3.5 at.% Y, 27 at.% Zr, 65 at.% O and 4 at.% Si. The superficial one (4) contains about 7 at.% Y, 21 at.% Si, 66 at.% O and 6 at.% P.

The data of the quantitative determination of the *t*-ZrO_2_ content in the PEM coatings are shown in Table 9.

According to the ZrO_2_-Y_2_O_3_ phase diagram, the yttria content of 6 mol.% in PEM coating with a thickness of 30 μm is quite sufficient for the *t*-ZrO_2_ phase formation. However, in reality, the mixture of *m*- and *t*-ZrO_2_ phases is observed. With coating thickness up to 80 μm elevation, the content of the *t*-ZrO_2_ phase increases. For full inhibition of the *m*-ZrO_2_ phase formation in PEM coating, Y_2_O_3_ content must exceed 8 mol.%. The needed conditions occur during modification in a slurry electrolyte with 6 g/L of yttria nanopowder, which leads to the formation of coatings with a thickness of 100 and 120 μm. The cubic *c*-ZrO_2_ cannot be formed according to the phase diagram of the ZrO_2_-Y_2_O_3_. The excess portion of the Y_2_O_3_ nanoparticles is not involved in the zirconia formation process and remains in the PEM coatings as inclusions.

Hence, the conclusion presented in [87] summarized that the formation of the *t*-ZrO_2_ phase in the PEM treatment of the Zr-1% Nb alloy in aqueous electrolytes containing 9 g/L sodium silicate and 5 g/L sodium hypophosphite is stimulated by the addition of yttria nanopowder. It has been suggested that during the processing, Y_2_O_3_ nanoparticles from the slurry electrolyte enter the plasma of micro-discharges and are involved in the formation of ZrO_2_-Y_2_O_3_ solid solutions. This is conditioned by the high local temperature (about several thousand Kelvin) in the zone of micro-discharge action. Dissolution of yttria depends not only on its concentration in the electrolyte, but also on the PEM coating thickness. The dissolution rate increases as the PEM coating thickness grows.

PEM of Zr followed by treatment in some substances allows obtaining anticorrosive superhydrophobic coatings [31]. Superhydrophobicity of the Zr samples treated in an aqueous solution containing 5 g/L of sodium aluminate was successfully reached by immersion in an ethanolic solution of stearic acid. A gradual increase in the contact angle was observed with the increase in treatment time in the solution. A maximum value of contact angle of 158° was obtained at a treatment time of 5 h. The morphological studies of PEM coatings reveal that the stearic acid treatment sealed the pores in the coatings. The superhydrophobic PEM layer exhibited the highest anticorrosion property—the corrosion current density was two orders of magnitude lower in the aqueous solution, containing 3.5% NaCl, than that of the PEM coating without treatment in the ethanolic solution of stearic acid.

Zirconium without a coating has very low bioactivity and does not lead to the formation of hydroxyapatite in simulated body fluid (SBF) [21]. However, Zr can be bioactivated by obtaining a porous PEM layer on its surface, and created coatings have a high potential for orthopedic implants. The process parameters have a huge impact on surface morphology and on the content and distribution of elements in the surface [32]. The surface of pure Zr was treated with PEM in an electrolyte containing 30 g/L calcium acetate monohydrate (Ca(CH_3_COO)_2_∙H_2_O), 8 g/L disodium beta glycerophosphate pentahydrate (C_3_H_7_Na_2_O_6_P∙5H_2_O) and 2 g/L anhydrous potassium hydroxide (KOH). By changing the process parameters, oxide coatings with different pore sizes were obtained. The results showed that the coatings comprised *t*-ZrO_2_ as well as a Ca_0.134_Zr_0.86_O_1.86_ phase as a product of interaction with electrolyte components. It was noted that when processing the Zr substrates, the surface roughness *Ra* was significantly influenced by the density and frequency of the anodic current. After holding the modified samples in SBF for 7 days, the formation of a hydroxyapatite structure on the surface was observed.

## 6. Conclusions

Thus, based on the presented analytical information review, it can be noted that zirconium and its alloys are increasingly used in various fields of activity. These materials are actively introduced in purely “peaceful” industries, primarily in medicine, especially in dentistry and orthopedics. Here, bio-tolerance and physical and mechanical properties play an important role. However, nuclear power and aerospace technology remain the main consumers of zirconium.

The most vulnerable point of zirconium and its alloys is insufficient resistance to environmental influences and changing thermodynamic conditions, even the most “soft”, not to mention aggressive and extreme ones. Surface engineering should be recognized as almost the only adequate solution that provides the required consumer properties. Of the whole range of surface modification methods, protective and functional ZrO_2_-based coatings provide the greatest efficiency.

Analysis and generalization of recent data and advances show that in modern practice, plasma electrolytic modification (PEM) or microarc oxidation (MAO) is the most rational among various methods of oxide-ceramic coat formation for products made of Zr and its alloys. This is connected with the optimal balance of technological, economic and target indicators, as well as the maximum universality of the results achieved. The given information and plasma electrolytic treatment modes can serve as a starting point when making decisions on a method for improving the product characteristics of zirconium material choice, or when carrying out relevant scientific and technological developments.

## Figures and Tables

**Table 1:** Strength characteristics of Zr and some of its alloys [43].

Material	Strength Indicator					
	20 °C			400 °C		
	σ_0.2_, MPa	σ_B_, MPa	δ, %	σ_0.2_, MPa	σ_B_, MPa	δ, %
Zr (iodide)	220	80	45	110	40	60
Zr (electrolytic)	855	230	36	155	65	52
Circaloy-2	310	480	22	70	170	36
Zr-1% Nb	200	350	30	90	180	38
Zr-2.5% Nb	280	450	25	180	270	22
E-125	446	456	31	238	272	34.6

**Table 2:** Physical and mechanical characteristics of ZrO_2_ [57,58,59,60].

Density, g/cm^3^	5.79–6.1
Flexural strength, MPa	700–2500
Young’s modulus, GPa	200–210
Crack resistance K_1C_, MPa∙m^1/2^	8–10
Vickers hardness, HV_0.1_, GPa	12–13
Thermal expansion coefficient, K^−1^∙10^6^	10.0–11.0
Coefficient of thermal conductivity, W/(m∙K)	2–3
Electrical conductivity, S∙m^−1^	2.1–2.4

**Table 3:** Treatment parameters for PEM coatings [77].

Composition (Na_2_SiO_3_ + KOH)	Sample No.	Current Density (A/cm^2^)	Treatment Time (min)
Group A Electrolyte I, pH = 11–12 (8 g/L + 0.8 g/L)	S1	0.05	4
	S2	0.1	2
	S3	0.25	0.8
Group B Electrolyte II, pH = 13–14 (8 g/L + 8 g/L)	S4	0.05	4
	S5	0.1	2
	S6	0.25	0.8

**Table 4:** Results of potentiodynamic polarization tests in a 0.2 mol/L LiOH solution [77].

Samples	i_corr_ (mA/cm^2^)	E_corr_ (V)	R_p_ (Ωcm^2^)
Zr-2.5Nb substrate	1.02 × 10^−3^	−0.35	1.22 × 10^4^
Black oxide coating	1.12 × 10^−5^	−0.28	3.57 × 10^6^
S1 (0.05 A/cm^2^, 8:0.8)	6.31 × 10^−5^	−0.25	1.22 × 10^6^
S2 (0.1 A/cm^2^, 8:0.8)	5.71 × 10^−5^	−0.27	1.39 × 10^6^
S3 (0.25 A/cm^2^, 8:0.8)	1.58 × 10^−5^	−0.22	1.58 × 10^6^
S4 (0.05 A/cm^2^, 8:8)	7.08 × 10^−5^	−0.25	5.72 × 10^5^
S5 (0.1 A/cm^2^, 8:8)	1.58 × 10^−4^	−0.21	2.68 × 10^5^
S6 (0.25 A/cm^2^, 8:8)	2.09 × 10^−4^	−0.28	2.06 × 10^5^

**Table 5:** Wear/failure distances of coated and uncoated samples under rotating mode [77].

Rotating Mode	Zr-2.5Nb	S1	S2	S3	S4	S5	S6
dry/2N/ v = 0.1 m/s	50 m	1000 m	970 m (failed)	1000 m	1000 m	1000 m	910 m (failed)

**Table 6:** Modes of formation and characteristics of PEM coatings [46].

Sample	U, V	Electrolyte Composition	τ, min	h, µm	P_g_, %	d, µm	λ_CM_, W/m·K	λ_coat_, W/m·K	M·10^4^, g/cm^2^
1	350	10% H_3_PO_4_	30	9	35.43	4.08	1.9666	0.086	3.176
2	300	10% H_3_PO_4_	60	15	31.75	6.05	1.6639	0.134	3.414
3	220	14 g/L Na_3_PO_4_	33	20	31.05	8.09	1.8278	0.175	1.985
		10% H_3_PO_4_	120						

**Table 7:** Values of the critical load when assessing the adhesive strength of coatings [80].

Electrolyte	Critical Load, N
APS	5.23 ± 0.57
AP	4.63 ± 0.56
AS	3.97 ± 0.57

**Table 8:** The chemical compositions of the electrolytes [84].

Electrolyte	A1	A2	A3	A4
Sodium silicate (g/L)	12	12	12	12
Yttrium acetate tetrahydrate (g/L)	0	1	2	4

**Table 9:** Ratio of tetragonal and monoclinic ZrO_2_ phases in PEM coatings [87].

Concentration of Y_2_O_3_ in Electrolyte, g/L	Duration of PEM Treatment, min	Coating Thickness, μm	*t*-ZrO_2_/(*m*-ZrO_2_+*t*-ZrO_2_), %
4	30	30	74
4	60	80	86
6	30	100	100
6	60	120	100

## Data Availability

All data presented in the review can be found at the appropriate links in the References section.

## Supplementary Materials

The following supporting information can be downloaded at: https://www.mdpi.com/article/10.3390/ma16165543/s1.
