# Supplementary material for: Plasma Electrolytic Modification of Zirconium and Its Alloys: Brief Review
Source: Materials (Basel). 2023 Aug 9;16(16):5543. doi: 10.3390/ma16165543 (PMC10456613; doi:10.3390/ma16165543)
Supplement: Supplementary file 1 [file materials-16-05543-s001.zip › materials-2308063-supplementary.pdf]

№ 335

ГОСУДАРСТВЕННЫЙ КОМИТЕТ РОССИЙСКОЙ ФЕДЕРАЦИИ
ПО ВЫСШЕМУ ОБРАЗОВАНИЮ

МОСКОВСКИЙ ГОСУДАРСТВЕННЫЙ ИНСТИТУТ
СТАЛИ И СПЛАВОВ
(ТЕХНОЛОГИЧЕСКИЙ УНИВЕРСИТЕТ)

Ракоч А.Г., Делян В.И., Васильев В.Ю., Казакевич А.В.

КОРРОЗИЯ И ЗАЩИТА МЕТАЛЛОВ

МОСКВА 1994

Их необходимость на данном этапе развития теории высокотемпературной коррозии металлов в газовой среде можно показать на следующем примере.

Рассматривая процесс высокотемпературного окисления циркония в газовой среде Фокин М.Н., Опара Б.К. и др. высказали рабочую гипотезу о том, что анодный процесс окисления циркония (и его аналогов: Ti , Hf) до диоксида является двухстадийным.

На первой стадии, реализующейся на границе раздела фаз "металл-оксидная пленка", имеет место образование монооксида циркония по реакции:

$$Zr + O^{2-} + 2\oplus \rightarrow ZrO \qquad (I.20)$$

На второй стадии, реализующейся вблизи границы $p - n$ -перехода, имеет место образование диоксида циркония по реакции

$$ZrO + O^{2-} + 2\oplus \rightarrow ZrO_2 \qquad (I.21)$$

Сопряженный катодный процесс протекает на внешней границе раздела фаз системы "цирконий- оксидная пленка-газ" по реакции

$$ZrO_2 + 1/2\,\delta O_2 + \delta\bar{e} \rightarrow ZrO_{2+\delta}^{\delta-} \qquad (I.22)$$

При этом они считают , что заторможенной (контролирующей) стадией всего процесса высокотемпературного окисления циркония в газовой среде является вторая стадия (стадия образования диоксида циркония по реакции (I.21) из-за низкой концентрации дырок $\oplus$ в n - проводящем слое оксидной пленки. К сожалению, ни электрографический, ни рентгенофазовый анализы, проведенные специально с этой целью, не подтвердили существование кубической фазы ZrO . Расчетные значения межплоскостных расстояний и интенсивностей хорошо соответствовали литературным данным для ZrO_2. Кроме того, Гельд П.В., Цхай Б.А. с сотрудниками, изучая влияние $Me_I - Me_{II}$ -взаимодействий на структурные и термодинамические свойства оксидов металлов IVа подгруппы, показали, что дефектность

в ZrO , HfO была бы больше 25%. Подобные высокодефектные оксиды не могут образовываться из-за их структурной неустойчивости.

Вместе с тем, пленка, формирующаяся при достаточно больших $P^i_{O_2}$ состоит из двух слоев: внутреннего слоя с недостатком кислорода по сравнению со стехиометрическим содержанием его в диоксиде циркония (n -проводящего слоя) и внешнего слоя - твердого раствора кислорода в ZrO_2 (например рис.I.12а).

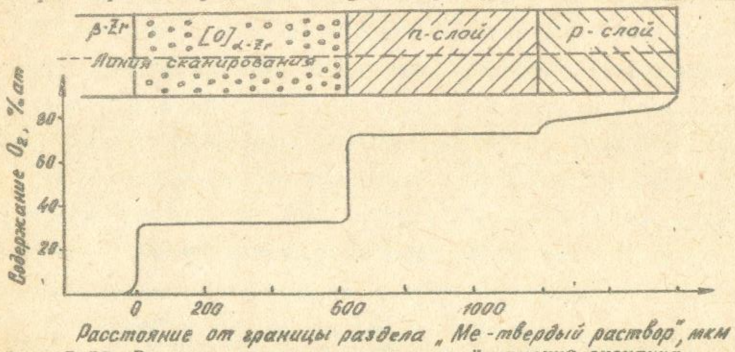

Рис. I.12а-Расположение слоев в системе "цирконий-оксидная пленка" и изменение концентрации кислорода по толщине слоев, сформированных после высокотемпературной (I670К) обработки металла при $P^i_{O_2} \approx 21280$ Па в течение 20 мин.

Учитывая, существование двух слоев в плёнке, Ракоч А.Г. с сотрудниками считали, что процесс взаимодействия циркония (а также титана и гафния) с кислородом может контролироваться заторможенностью реакции

$$MeO_{2-\delta} + (\delta + k)O^{2-} + 2(k+\delta)\oplus \rightarrow [O]_{MeO_2} , \qquad (I.23)$$

где δ - недостаток, а k - избыток кислорода по сравнению со стехиометрическим содержанием его в диоксиде металла .

Однако, контролирующей стадией процесса высокотемпературно-

$R_п^\Sigma$ является функцией от вида поляризации системы. При обратном смещении $p-n$ -перехода (анодная поляризация) и при отсутствии пробоя $p-n$ -перехода сумма этих сопротивлений, реализующихся на границах раздела слоев из продуктов взаимодействия металла с кислородом, должна увеличиваться, а $J_{изм}$ - уменьшаться с увеличением U_{12} и, наоборот, $R_п^\Sigma$ -уменьшаться, а $J_{изм}$ - увеличиваться при прямом смещении $p-n$ -перехода (катодная поляризация этой же системы) с увеличением U_{12}.

При небольшой изотермической выдержке и $U_{12} \approx 4 \cdot 10^{-2}$В, экспериментальные результаты, приведенные на рис.2.8 подтверждает это.

Однако, если при анодной поляризации системы "цирконий - пленка-газ" произойдет пробой $p-n$ -перехода и внешнего слоя пленки, то из-за явления "ударной ионизации" произойдет лавинное **умножение носителей** (электронов и дырок). Обратный ток (поток электронов) через $p-n$ -переход и **внешний** слой пленки начнет чрезвычайно резко возрастать с дальнейшим ростом напряжения (U_{12}). При возрастании обратного напряжения (по отношению к $p-n$ -переходу) на несколько процентов ток (поток электронов) через переход и внешний слой мог бы возрасти в сотни тысяч и даже миллионы раз, т.е. при пробое $p-n$ -перехода и p -слоя их сопротивления ($R_э^p$ и $R_э^{p-n}$) следует рассматривать как стремящиеся к нулю.

Учитывая, что сумма остальных электронных сопротивлений слоев из продуктов взаимодействия циркония с кислородом стремится к нулю, то при пробое $p-n$ -перехода и внешнего слоя пленки уравнение (2.18) существенно упрощается -

$$J^A_{изм} = \frac{E_{внеш}}{RT}, \qquad (2.22)$$

т.е. $J^A_{изм}$ и должно стать намного больше $J^K_{изм}$, при тех же внешних условиях ($P^i_{O_2}$, T) и $E_{внеш} \geqslant 4 \cdot 10^{-2}$В и $\tau > \tau_{крат}$ (рис.2.8).

Пробой внешнего слоя пленки подтверждается визуально наблюдаемыми нарушениями сплошности этого слоя. Формирующийся при высокотемпературном окислении циркония при $P^i_{O_2} \approx 21280$Па в пленке $p-n$ -переход характеризуется низкими значениями (рис.2.8).

Увеличение (рис. 2.9) $E_{внеш}$ (до I-5В) приводит к более значительному увеличению $K^-_{асим}$ при всех длительностях изотермической выдержки.

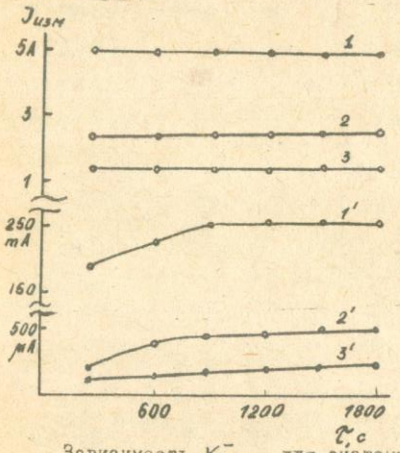

Рис.2.9 Зависимость от длительности изотермической (I570K) выдержки при анодной (I,2,3) и катодной (I^I, 2^I, 3^I) поляризации системы "цирконий - двухслойная оксидная пленка" при $P^i_{O_2} \approx 21280$Па и $E_{внеш}$:
I, I^I-5; 2,2^I-2; 3,3^I-IB.

Таблица 2.2.

Зависимость $K^-_{асим}$ для системы "цирконий-двухслойная пленка" с подключенным токоизмерительным прибором при T = I570K, $P^i_{O_2} \approx 21280$ Па от $E_{внеш}$. при различных τ.

Eвнеш. , В	τ , с	K⁻асим.
I	2	3
I	300	25,4
